# Identification of distinct epitopes in dipeptidyl peptidase-4 inhibitor–associated bullous pemphigoid

**DOI:** 10.1126/sciadv.adv9423

**Published:** 2025-08-01

**Authors:** Shoko Mai, Yosuke Mai, Inkin Ujiie, Kentaro Izumi, Ken Natsuga, Wataru Nishie, Hideyuki Ujiie

**Affiliations:** Department of Dermatology, Faculty of Medicine and Graduate School of Medicine, Hokkaido University, Sapporo, Japan.

## Abstract

Bullous pemphigoid (BP) is a common autoimmune skin disorder caused by autoantibodies targeting BP180. Recent evidence shows that dipeptidyl peptidase-4 inhibitors (DPP4i), used in diabetes management, can induce BP (DPP4i-BP). DPP4i-BP differs from typical BP in its genetic, clinical, and immunological features, but methods to specifically detect DPP4i-BP autoantibodies have been unavailable. This study used enzyme-linked immunosorbent assay with “domain-swapped BP180” proteins to identify autoantibodies in DPP4i-BP, which targeted BP180 regions from the seventh noncollagenous domain to the fourth collagenous domain (NC7-Col4). These epitopes were associated with DPP4i-BP–specific human leukocyte antigen class II peptide epitopes. Notably, the duration of DPP4i intake before BP onset was significantly shorter in patients with anti–NC7-Col4 autoantibodies than those without them. Measuring the autoantibodies enabled the early diagnosis of DPP4i-BP before epitope spreading. Furthermore, anti–NC7-Col4 autoantibodies were detected in some patients with diabetes taking DPP4i without BP, suggesting that these assays may offer potential tools for early identification of at-risk individuals.

## Teaser

Domain-swapped BP180 ELISA identifies DPP4i-BP–specific autoantibodies, allowing early diagnosis and distinction from typical BP.

## INTRODUCTION

Bullous pemphigoid (BP) is the most prevalent autoimmune blistering disease, characterized by tense blister formation on urticarial erythema across the body (*1*–*4*). BP predominantly affects older people, in whom it has high mortality; moreover, its prevalence is increasing in aging societies (*5*), underscoring the need for understanding and management of BP. The pathogenesis of BP primarily involves autoantibodies targeting hemidesmosomal components in basal keratinocytes: BP180 (BPAG2 or type XVII collagen) and BP230 (BPAG1) (*1*, *6*–*9*). BP180 is a type II transmembrane collagen protein consisting of 15 collagenous (Col) domains in its extracellular domain, interspersed with noncollagenous (NC) domains (*10*, *11*), which forms a triple helix structure. Notably, 80 to 90% of these autoantibodies react to the NC16A domain of BP180 (*1*, *3*, *12*, *13*). The enzyme-linked immunosorbent assay (ELISA), using the NC16A domain of BP180, plays a significant role in BP diagnosis, enabling clinical severity assessment and disease activity monitoring (*14*).

However, targeting regions outside the NC16A domain by anti-BP180 autoantibodies is an intriguing aspect of BP pathogenesis (*12*). A subset of patients with BP has autoantibodies targeting regions of BP180 outside the NC16A domain, often presenting with milder disease severity and fewer erythema than typical BP cases with anti-BP180 NC16A autoantibodies. In addition, on the basis of the following human and murine studies, the presence of anti-BP180, non-NC16A autoantibodies might reflect the preclinical condition of BP. Epidemiological data showed that 2.2% of healthy individuals harbor circulating anti-BP180, non-NC16A autoantibodies without BP development (*15*). In mice, a germ line–encoded anti-BP180 antibody is also directed against the non-NC16A region (*16*). In mucous membrane pemphigoid, autoantibodies targeting C-terminal BP180 epitopes are strongly associated with predominant mucosal involvement, demonstrating that epitope specificity drives distinct clinical phenotypes (*17*–*19*). Moreover, low-level anti-NC16A reactivity is sometimes detected in patients with other pruritic dermatoses, such as eczema or urticaria, underscoring the need for clinical correlation when interpreting NC16A ELISA results (*20*–*22*). These observations raise the hypothesis that the presence of anti-BP180, non-NC16A autoantibodies may be a potential risk factor for BP development.

Another aspect of the pathogenesis of anti-BP180, non-NC16A autoantibodies is drug-induced BP. Recent evidence has revealed that the use of dipeptidyl peptidase-4 inhibitors (DPP4i), a common medication for type II diabetes mellitus (DM), is associated with BP development, namely DPP4i-associated BP (DPP4i-BP) (*23*–*28*). Although the reported clinical and immunological features of DPP4i-BP vary among studies (*25*–*30*), a specific group of patients with DPP4i-BP primarily has anti-BP180, non-NC16A autoantibodies, manifesting a “noninflammatory” phenotype that exhibits less erythema than in typical BP (*13*, *30*–*32*). Previously, we demonstrated that these autoantibodies preferentially react to the midportion of the BP180 extracellular domain (*33*), highlighting the specific epitopes differentiating from the epitopes of typical BP; however, the specific epitope has not yet been identified. A total of 10.9% of patients with DM treated with DPP4i has anti-BP180, non-NC16A autoantibodies without clinical BP manifestation (*34*). In addition, continued use of DPP4i after BP onset may exacerbate disease activity, leading to increased erythema and epitope spreading of autoantibodies from the non-NC16A to NC16A region (*35*, *36*). These studies suggest that the specific anti-BP180, non-NC16A autoantibodies, distinct from the autoantibodies of typical BP, are primarily present in the preclinical or early stages of DPP4i-BP. Here, we aim to identify the primary epitope targeted by DPP4i-BP autoantibodies to understand the pathomechanism of DPP4i-BP and improve the diagnostic approach and therapeutic strategies for DPP4i-BP.

## RESULTS

### Differential reactivity of DPP4i-BP autoantibodies to the conformational structures of BP180

**Fig. 1.**
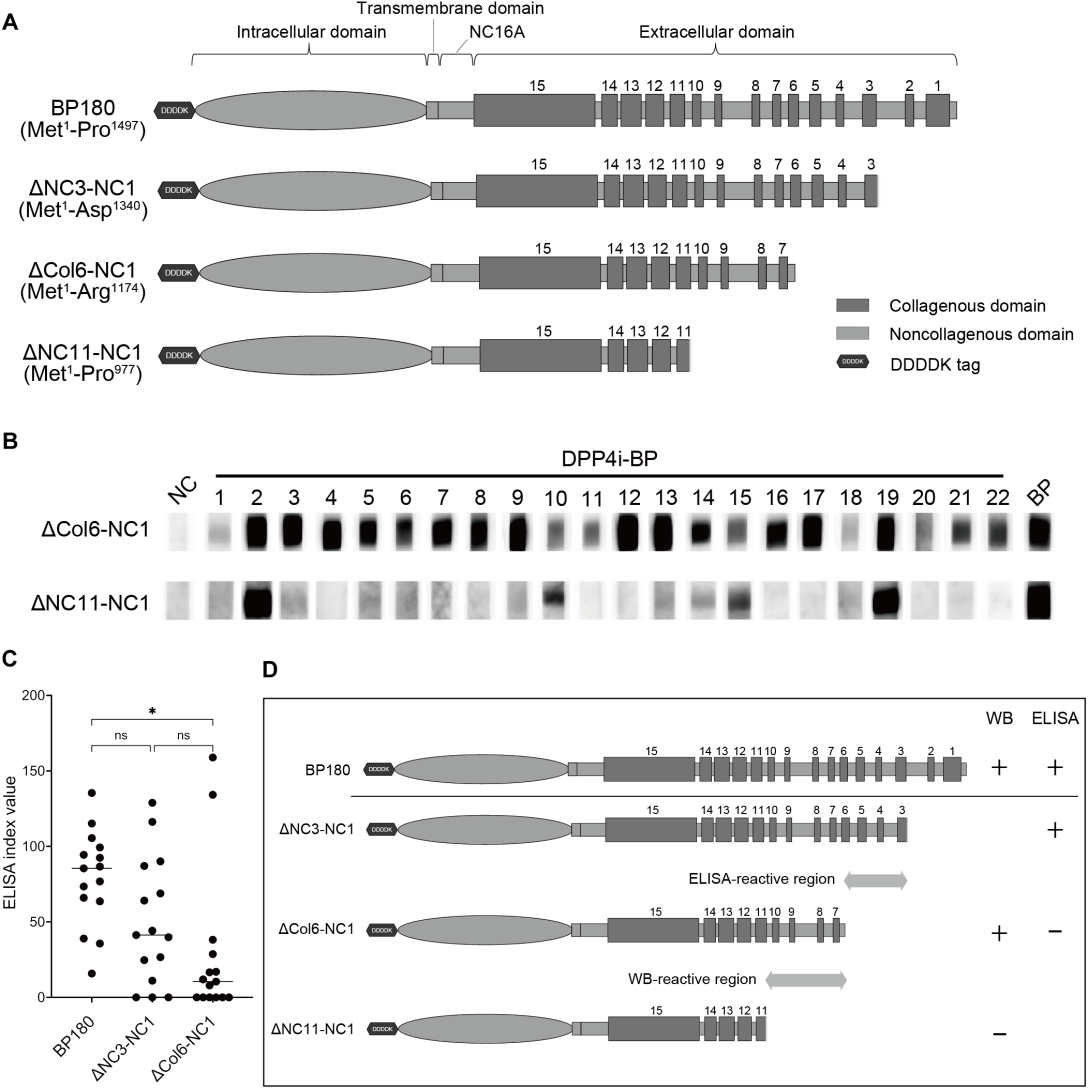
Epitope mapping of NC16A(−) DPP4i-BP using truncated forms of BP180. (**A**) Schematic of full-length and truncated forms of recombinant BP180. (**B**) WB with patient sera for the full-length and each truncated form of recombinant BP180. (**C**) ELISA with patient sera for the full-length and each truncated form of recombinant BP180. Statistical analysis was performed using Dunn’s multiple comparisons test. ns, not significant. 0.01 < *P < 0.05. (**D**) Schematic of the respective epitopes identified by ELISA and WB.

We first selected DPP4i-BP cases with anti-BP180, non-NC16A autoantibodies, referred to as NC16A(−) DPP4i-BP (fig. S1 and table S1). We analyzed the epitopes of NC16A(−) DPP4i-BP autoantibodies using a series of sequential C-terminal truncated recombinant BP180 proteins: full-length: Met^1^-Pro^1497^, ΔNC3-NC1: Met^1^-Asp^1340^, ΔCol6-NC1: Met^1^-Arg^1174^, and ΔNC11-NC1: Met^1^-Pro^977^ (Fig. 1A). In Western blotting (WB), all NC16A(−) DPP4i-BP sera reacted with the ΔCol6-NC1 truncated protein, while only six sera showed reactivity to the ΔNC11-NC1 protein (Fig. 1B). This suggests that a significant proportion—at least 73%—of NC16A(−) DPP4i-BP sera targets epitopes containing Ser^978^-Pro^1174^, corresponding to the NC11-NC7 region of BP180, in WB.

Subsequently, the same 22 NC16A(−) DPP4i-BP sera were tested in ELISA using full-length BP180, ΔNC3-NC1, and ΔCol6-NC1 as substrates. All NC16A(−) DPP4i-BP sera demonstrated positive reactivity with the full-length BP180 in ELISA. The comparative analysis between full-length BP180 and ΔNC3-NC1 revealed no significant difference in seroreactivity. However, the reactivity toward ΔCol6-NC1 was unexpectedly lower, indicating that NC16A(−) DPP4i-BP autoantibodies reacted to epitopes located in Gly^1175^-Asp^1340^, corresponding to the Col6-Col3 regions of BP180 in ELISA (Fig. 1C). Because the estimated epitopes of NC16A(−) DPP4i-BP autoantibodies differed between WB and ELISA (Fig. 1D), we hypothesized that this could be caused by the conformational alteration of the BP180 protein: The ELISA used native trimeric recombinant BP180 proteins, whereas WB used denatured monomeric forms.

### Formation of the Col trimer by domain-swapped BP180 proteins

**Fig. 2.**
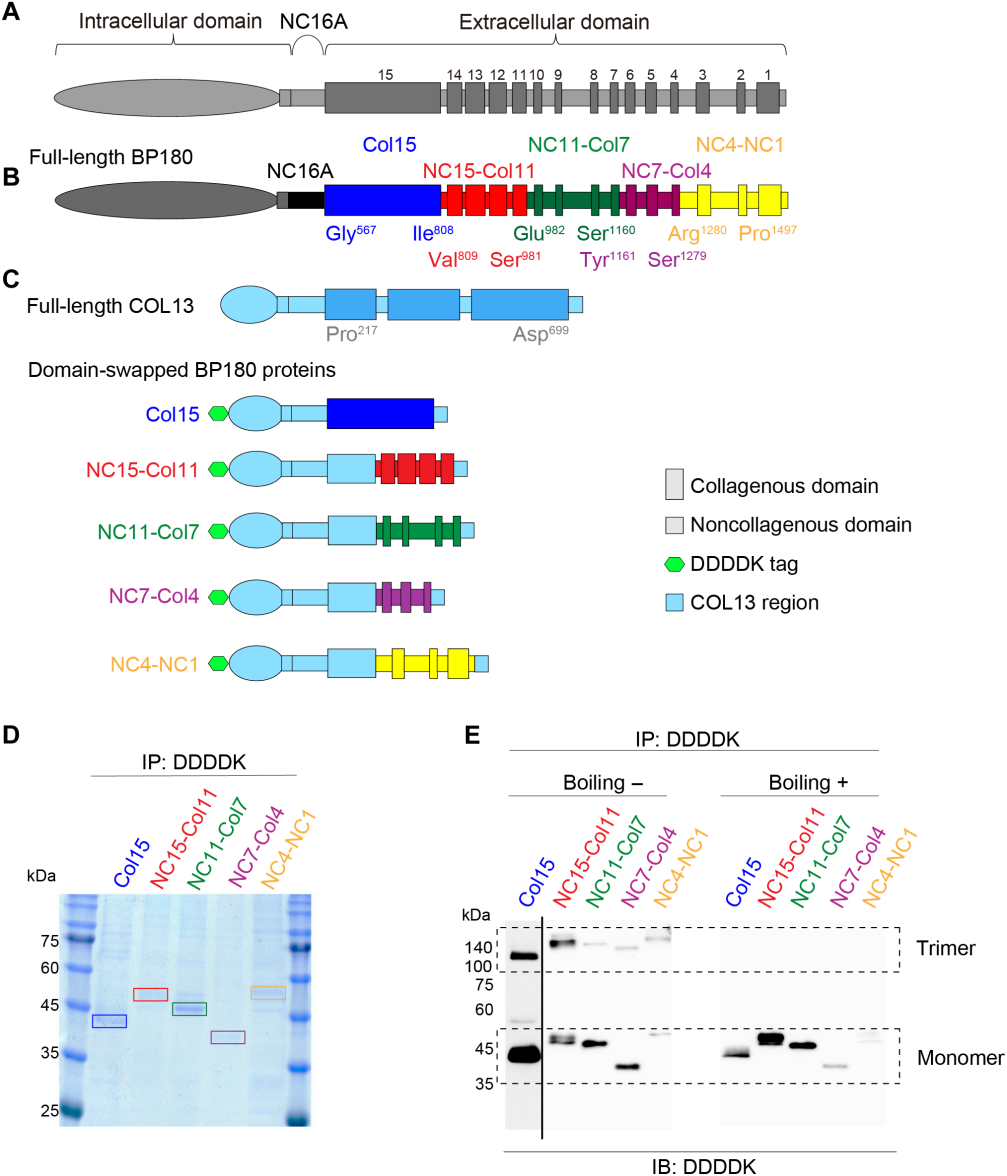
Characterization of domain-swapped BP180. (**A**) Structure of the full-length BP180. (**B**) The extracellular domain of BP180 was divided into five fragments. (**C**) Structures of the full-length COL13 and each of the domain-swapped BP180 recombinant proteins. Each of the domain-swapped BP180 proteins has a DDDDK tag at its N terminus. COL13 is used as the backbone, and each domain-swapped protein has a fragment of the BP180 extracellular region. (**D**) Coomassie Blue staining of the domain-swapped BP180. IP, immunoprecipitation. (**E**) Immunoblotting (IB) of the domain-swapped BP180 using an anti-DDDDK mouse monoclonal antibody. In the nonboiled states, they showed bands that matched the molecular weight of each protein and bands that were three times larger. In the boiled samples, one band was observed for each protein, corresponding to the molecular weight of each protein.

The pilot analysis that implied conformational BP180 epitopes in NC16A(−) DPP4i-BP motivated us to use a domain-swapping approach for conformational epitope mapping. This method allows shorter constructs while maintaining protein conformation and was previously used in the epitope-mapping study of pemphigus vulgaris, another autoimmune blistering disease (*37*). In our study, we used collagen XIII (COL13), a type II transmembrane collagen that shares a similar trimer structure with BP180, and engineered five recombinant molecules, each representing a distinct conformational region of the BP180 extracellular domain swapped with COL13 (*38*). These regions included 15 Col and 15 NC domains (Fig. 2A), excluding the NC16A domain, and were strategically divided to avoid the disruption of their domain structures (Fig. 2B). The domain-swapped molecules were expressed in the Flp-In 293 expression system and were successfully purified by immunoprecipitation using the DDDDK tag (Fig. 2, C and D). We verified the ability of these domain-swapped proteins to maintain a trimeric conformation under nonboiling conditions (Fig. 2E).

### DPP4i-BP autoantibodies preferentially reacted to the NC7-Col4 region of BP180 in ELISA

**Fig. 3.**
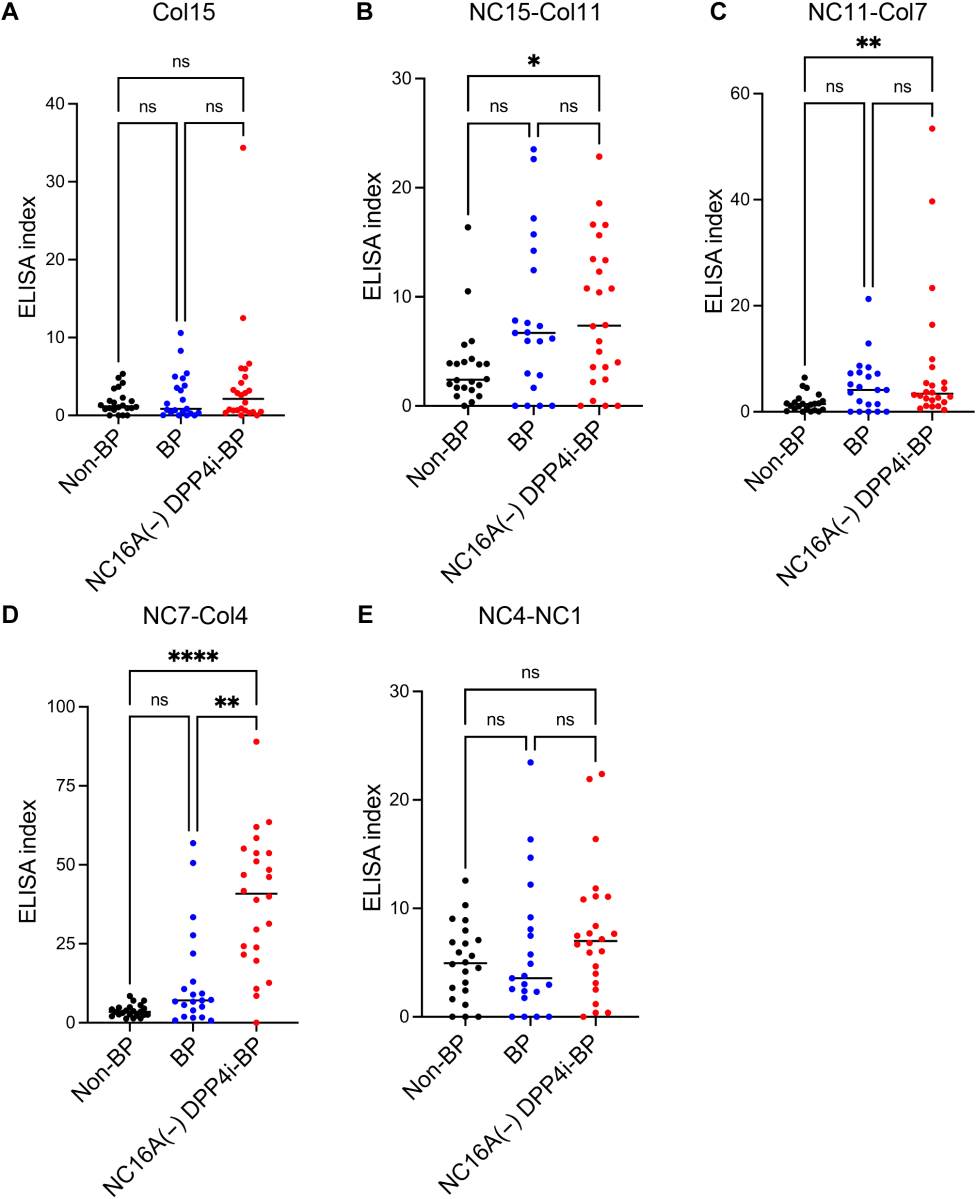
Domain-swapped BP180 ELISAs. (**A** to **E**) Scatter plot of each ELISA. Statistical analysis was performed using Dunn’s multiple comparisons test. 0.01 < *P < 0.05; 0.001 < **P < 0.01; ****P < 0.0001. [(A), (B), and (E)] NC16A(−) DPP4i-BP sera showed no difference against Col15, NC15-Col11, and NC4-NC1 compared to BP and non-BP sera. (C and D) NC16A(−) DPP4i-BP sera showed a predominant response to NC11-Col7 and NC7-Col4 compared to BP and non-BP sera.

For epitope mapping of anti-BP180 autoantibodies in NC16A(−) DPP4i-BP sera, we prepared ELISAs using each of the domain-swapped BP180 proteins as substrates. We performed domain-swapped BP180, NC16A, and full-length BP180 ELISA using NC16A(−) DPP4i-BP, BP, and non-BP control sera (Fig. 3, A to E, fig. S1, and tables S2 and S3). The results showed that NC16A(−) DPP4i-BP sera significantly reacted to NC15-Col11–, NC11-Col7–, and NC7-Col4–swapped proteins but not to Col15- or NC4-NC1–swapped proteins (Fig. 3, A to E). NC16A(−) DPP4i-BP sera predominantly reacted to the NC7-Col4–swapped protein compared to BP sera (Fig. 3D), indicating that DPP4i-BP has specific epitopes in the NC7-Col4 region that can differentiate DPP4i-BP from BP. At a cutoff value of 8.485 from the receiver operating characteristic curve, the NC7-Col4 ELISA had a sensitivity of 96% and a specificity of 100% for NC16A(−) DPP4i-BP versus non-BP sera (fig. S2). Thus, the DPP4i-BP autoantibodies preferentially reacted to the NC7-Col4 regions of BP180 in ELISA.

### DPP4i-BP autoantibodies preferentially reacted against the NC11-Col7 region of BP180 in WB

**Fig. 4.**
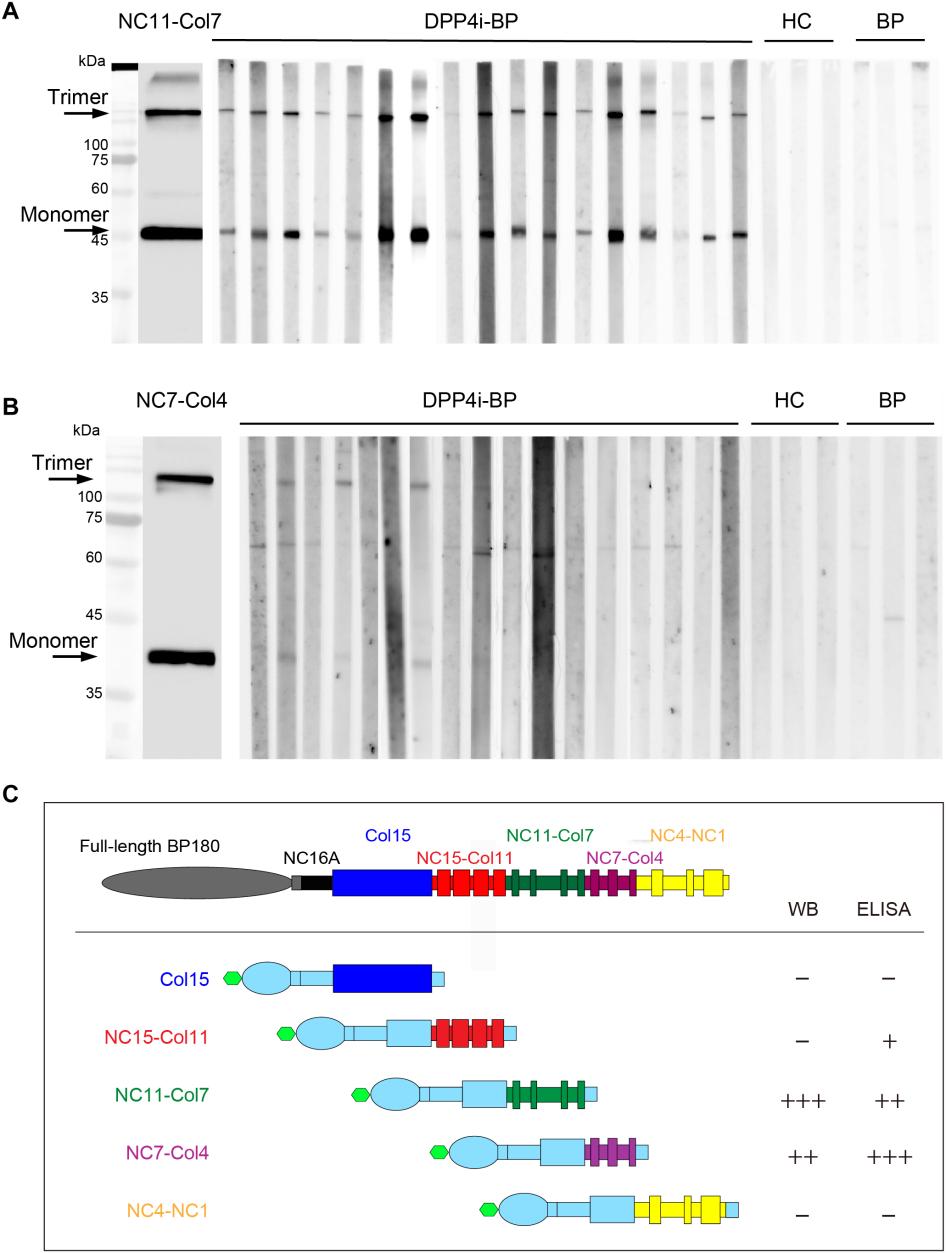
WB with patient sera for each domain-swapped BP180 recombinant protein. (**A**) Against NC11-Col7, all NC16A(−) DPP4i-BP sera were reacted with a trimer and monomer. (**B**) Four of 17 NC16A(−) DPP4i-BP sera responded to NC7-Col4. (**C**) Schematic diagram demonstrating reactivity in ELISA and WB with domain-swapped BP180 recombinant protein.

As the pilot study showed that the ELISA-reactive epitopes were different from WB-reactive ones (Fig. 1, A to D), we asked whether the reactivity of domain-swapped protein could also be altered in WB compared with ELISA. Unlike the ELISA data (Fig. 3C), WB showed that all NC16A(−) DPP4i-BP sera reacted with NC11-Col7 trimers and monomers (Fig. 4A). Some DPP4i-BP sera reacted with the trimeric (7 of 17) and monomeric forms of NC7-Col4 (4 of 17) (Fig. 4B), which also contrasted with the predominant reactivity of NC7-Col4 in the ELISA system (Fig. 3D). None of the three BP sera and healthy control sera reacted with NC11-Col7 or NC7-Col4 (Fig. 4, A and B). No autoantibodies were detected against Col15, NC15-Col11, or NC4-NC1 (fig. S3, A to C). From these data, NC16A(−) DPP4i-BP autoantibodies detected by WB mainly target the NC11-Col7 region of BP180. This discrepancy between ELISA and WB recapitulated the results of sequential C-terminal truncated BP180 proteins (Fig. 1, B to D), further corroborating the presence of conformational epitopes in BP180 (Fig. 4C).

### Primary epitope prediction of DPP4i-BP autoantibodies using DPP4i-BP dominant HLA classes

**Fig. 5.**
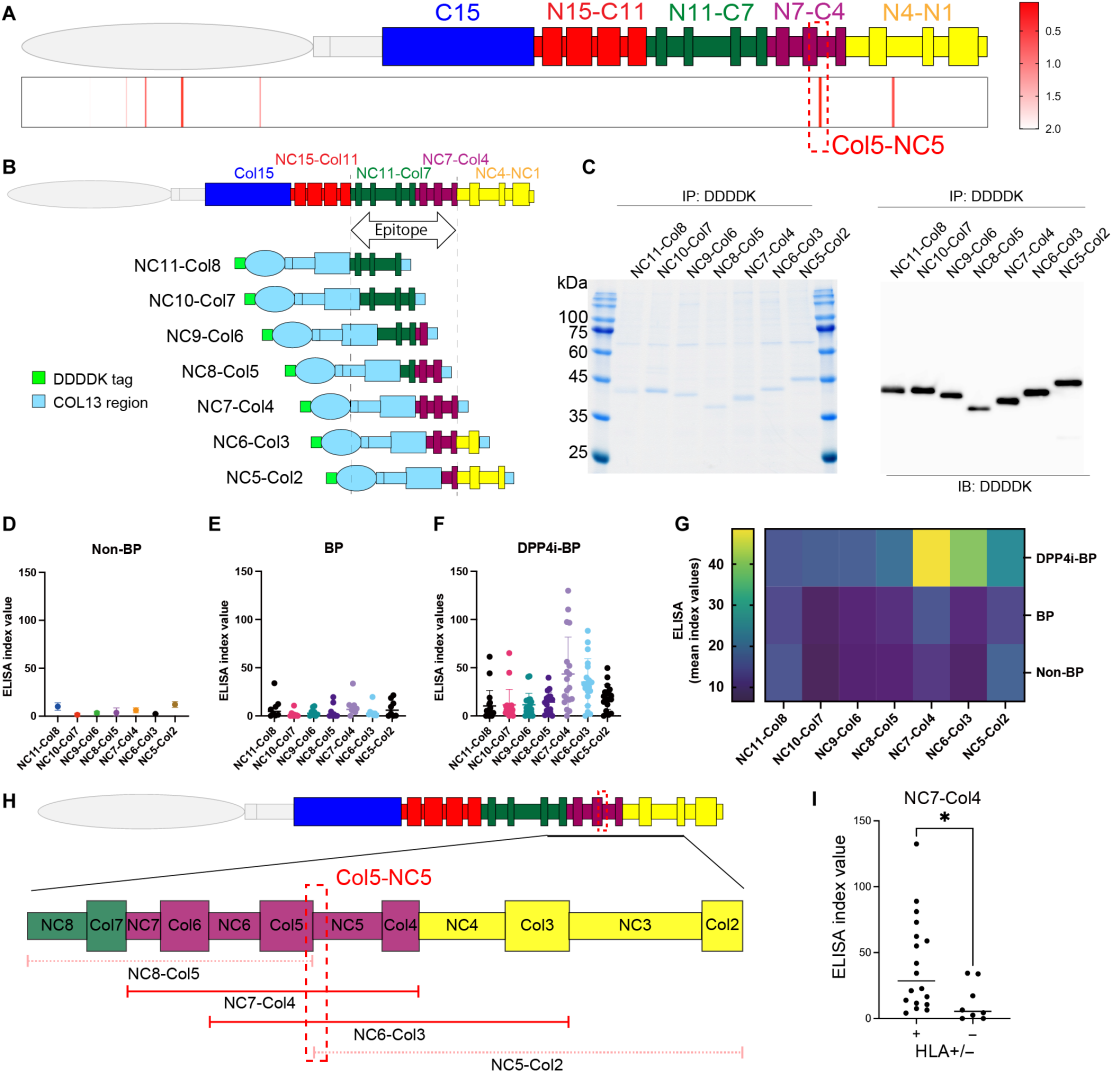
Characterization of shift domain-swapped BP180. (**A**) Distribution of high-binding-affinity site candidates in the BP180 full-length protein sequence of the DQA1*05:05 and HLA-DQB1*03:01 pair. Red bars represent strong-binding-affinity epitopes with <2% rank. (**B**) Structural diagram of “shift domain-swapped BP180” recombinant proteins, created by shifting one NC region and one collagen region in the NC11 to Col2 region. (**C**) Coomassie Blue staining and WB with an anti-DDDDK mouse monoclonal antibody for shift domain-swapped BP180. (**D** to **F**) Scatter plot of shift domain-swapped BP180 ELISA. DPP4i-BP sera showed increased index values for NC7-Col4, NC6-Col3, and NC5-Col2, while BP and non-BP sera showed no increase for any shift domain-swapped BP180 protein. (**G**) Heatmap diagram showing that most DPP4i-BP sera reacted with NC7-Col4 and NC6-Col3 in ELISA using shift domain-swapped BP180 recombinant proteins. (**H**) Schematic of a zoomed-in view of the BP180 extracellular domain sequence from NC8 to Col2 for epitope focusing. Red bars indicate the range contained in each shifted domain-swapped protein, with NC7-Col4 and NC6-Col3, which showed a response, shown as solid red lines and NC8-Col5 and NC5-Col2, which showed a reduced response, shown as pale red dashed lines. (**I**) Comparison of patients with DPP4i-BP who have HLA-DQA1*05 and HLA-DQB1*03:01 (shown as +) with patients with DPP4i-BP who do not have these HLAs (shown as −) using NC7-Col4 ELISA. 0.01 < *P < 0.05.

Our data identified two major epitopes of DPP4i-BP: NC11-Col7 and NC7-Col4, identified by WB and ELISA, respectively. To determine which of the two epitopes is more relevant to DPP4i-BP, we focused on the genetic background of DPP4i-BP. We previously reported that HLA-DQA1*05 and HLA-DQB1*03:01 are common haplotypes in Japanese patients with DPP4i-BP (*13*, *39*), although the mechanism of human leukocyte antigen’s (HLA’s) contribution to the pathogenesis of DPP4i-BP is largely unknown. We hypothesized that the HLA-DQ pair could present the specific region(s) of BP180, eventually resulting in the development of anti-BP180 autoantibodies. Therefore, we screened HLA class II peptide epitopes of BP180 with high affinity for HLA-DQA1*05:05 and HLA-DQB1*03:01 using the netMHCIIpan4.3 algorithm (*40*). The results showed that seven peptide epitopes with core peptides ranked in the top 2% were found in BP180 (Fig. 5A and table S4). The peptide with the highest % rank was PPGVSGALATYAAEN (core peptide: VSGALATYA) with % rank = 0.02 located at the Col5-NC5 boundary region, which was included in the NC7-Col4 protein (Fig. 5A and table S4).

To further clarify whether the HLA-predicted region is the primary epitope in DPP4i-BP, we generated domain-swapped BP180 proteins using the region from NC11 to Col2, shifting one NC domain and one Col domain together as one block; we named this “shift domain-swapped BP180” (Fig. 5B). Expression of shift domain-swapped BP180 proteins was confirmed by Coomassie Brilliant Blue staining and WB (Fig. 5C). ELISA using these shift domain-swapped BP180 proteins showed that DPP4i-BP sera predominantly reacted with the domain-swapped BP180 NC7-Col4 and NC6-Col3 proteins, while no reaction was observed with non-BP and BP sera (Fig. 5, D to G). As the reactivity of DPP4i-BP sera was reduced in ELISA using NC8-Col5 or NC5-Col2, the epitopes containing the Col5-NC5 region may be the primary antigenic region of DPP4i-BP (Fig. 5H), which is in line with our in silico analysis (Fig. 5A). HLA typing was performed in 26 newly diagnosed patients with DPP4i-BP enrolled between 2015 and 2018, all of whom also underwent domain-swapped ELISA (table S5). These 26 individuals represent a subset of the 60 patients listed in table S6. Notably, all patients carried either both HLA-DQA1*05 and HLA-DQB1*03:01 or neither; no cases had only one of the two alleles. On the basis of this distribution, we classified patients into two groups, HLA (+) (both alleles present) and HLA (−) (both alleles absent), and compared their autoantibody profiles accordingly. We also found that patients in the HLA (+) group exhibited significantly higher reactivity to the NC7-Col4 region compared to those in the HLA (−) group (*P* < 0.05) (Fig. 5I). These data imply that the NC7-Col4 region of BP180 might be the main epitope in DPP4i-BP, possibly through the specific HLA affinity for the conformation of the peptides.

### Characteristics of DPP4i-BP with anti-BP180 NC7-Col4 autoantibodies

**Fig. 6.**
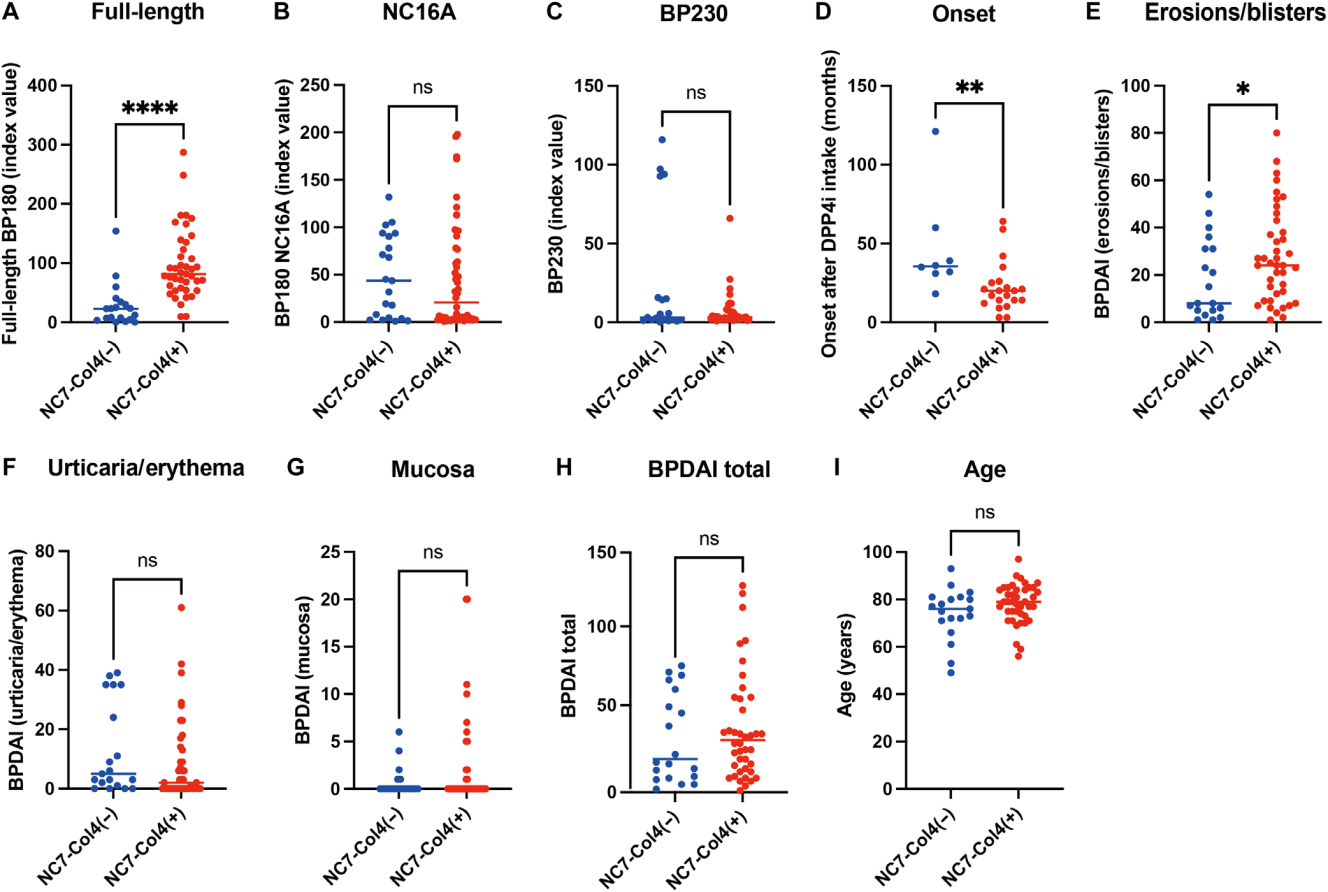
Characteristics of patients with DPP4i-BP who have and do not have anti-BP180 NC7-Col4 autoantibodies. (**A** to **I**) Scatter plot comparing patients with DPP4i-BP who have and do not have NC7-Col4 autoantibodies for each factor. (A, D, and E) NC7-Col4(+) DPP4i-BP had higher full-length BP180 index values, shorter time from DPP4i oral initiation to onset, and higher BPDAI scores for erosions/blisters than NC7-Col4(−) DPP4i-BP. (B, C, and F to I) However, there were no significant differences in NC16A and BP230 ELISA index values, BPDAI scores for urticaria/erythema, mucosa, total BPDAI score, and age. 0.01 < *P < 0.05; 0.001 < **P < 0.01; ****P < 0.0001.

Despite the predominant reactivity of the NC7-Col4 epitope, not all DPP4i-BP autoantibodies reacted with this region (Figs. 3D and 5F). We wondered whether there were immunological and phenotypical differences between patients with DPP4i-BP who have anti-BP180 NC7-Col4 autoantibodies and those who do not. To elucidate this, we retrospectively examined the sera of patients with DPP4i-BP collected in our department from 2013 to 2022. The analyzed characteristics of patients with DPP4i-BP are shown in table S6. In this population, 93.3 and 68.3% of DPP4i-BP cases were positive for full-length BP180 ELISA and BP180 NC7-Col4–swapped ELISA, respectively, while 66.7% were positive for BP180 NC16A ELISA. Simple linear regression analysis revealed that index values of full-length BP180 ELISA were significantly correlated with those of BP180 NC7-Col4–swapped ELISA, while index values of full-length BP180 ELISA and BP180 NC16A ELISA were not (fig. S4, A and B), suggesting that the conformation of the NC7-Col4 region in the full-length BP180 is preserved in the swapped protein. Then, we classified this population into DPP4i-BP with or without anti-BP180 NC7-Col4 autoantibodies. In accordance with the simple linear regression analysis (fig. S4A), NC7-Col4(+) DPP4i-BP exhibited significantly higher median index values of full-length BP180 (median: 81.6 versus 22.7) and positive rates (100% versus 78.9%) compared to NC7-Col4(−) DPP4i-BP, with *P* values of <0.0001 and 0.0079, respectively (Fig. 6A and Table 1). In contrast, index values and positive rates of BP180 NC16A ELISA and BP230 ELISA did not significantly differ between NC7-Col4(+) DPP4i-BP and NC7-Col4(−) DPP4i-BP (Fig. 6, B and C, and Table 1). Among DPP4i drugs, linagliptin was used most frequently in NC7-Col4(+) DPP4i-BP, followed by teneligliptin and vildagliptin (Table 1). In contrast, sitagliptin was least used in NC7-Col4(+) DPP4i-BP (*P* = 0.0431; odds ratio: 0.2; 95% confidence interval: 0.1 to 1.0) (Table 1). Unexpectedly, the duration of DPP4i intake before DPP4i-BP onset was shorter in NC7-Col4(+) DPP4i-BP than in NC7-Col4(−) DPP4i-BP, with median duration values of 20.0 and 35.5 months, respectively (*P* = 0.0028) (Fig. 6D and Table 1). These data suggest that patients with DM who produce anti-BP180 NC7-Col4 autoantibodies could develop BP sooner after the use of DPP4i compared to those who do not harbor autoantibodies for that region.

**Table 1.** Comparison between DPP4i-BP NC7-Col4(−) and NC7-Col4(+). OR, odds ratio; CI, confidence interval. Bolded values indicate statistically significant differences (*P* < 0.05).

Characteristics	NC7-Col4			
	Negative (*n* = 19)	Positive (*n* = 41)	*P* value	OR (95% CI)
Age, median (range), years	76 (49–93)	79 (56–97)	0.1500	
Female sex, *n* (%)	6 (31.5)	17 (41.5)	0.5729	1.5 (0.5–5.2)
Full-length BP180				
Median (range), index value	22.7 (0.0–154.1)	81.6 (9.6–287.1)	**<0.0001**	
Positive cases (%)	15 (78.9)	41 (100)	**0.0079**	∞ (2.3–∞)
BP180 NC16A				
Median (range), index value	45.0 (0.9–131.8)	34.7 (1.3–198.1)	0.7083	
Positive cases (%)	14 (73.7)	26 (63.4)	0.5601	
BP230				
Median (range), index value	3.0 (0.0–115.8)	3.3 (0.9–65.9)	0.9969	
Positive cases (%)	7 (36.8)	7 (17.1)	0.1108	0.4 (0.1–1.2)
DPP4i, *n* (%)				
Vildagliptin	6 (24.0)	9 (19.6)	0.7632	0.8 (0.2–2.7)
Sitagliptin	7 (28.0)	4 (8.7)	**0.0431**	0.2 (0.1–1.0)
Linagliptin	3 (12.0)	16 (34.8)	0.0505	3.9 (1.1–14)
Teneligliptin	5 (20.0)	10 (21.7)	>0.9999	1.1 (0.3–3.4)
Alogliptin	3 (12.0)	6 (13.0)	>0.9999	1.2 (0.3–4.8)
Trelagliptin	1 (4.0)	0 (0.0)	0.3521	0.0 (0.0–4.9)
Anagliptin	0 (0.0)	1 (2.2)	>0.9999	∞ (0.1–∞)
Onset after DPP4i intake (*n* = 8 versus 21), median, months	35.5 (7–121)	20.0 (3–64)	**0.0028**	
BPDAI, median (range), score				
Erosions/blisters	8 (1–54)	24 (1–80)	**0.0458**	
Urticaria/erythema	5 (0–39)	2 (0–61)	0.1559	
Mucosa	0 (0–6)	0 (0–20)	0.6012	
Total	19 (2–75)	31 (1–129)	0.4608	
Without oral PSL (*n* = 17 versus 39), positive rate (%)	41.2	48.7	0.7719	1.5 (0.5–4.5)

We also compared the disease severity between NC7-Col4(+) and NC7-Col4(−) DPP4i-BP. The scores for erosions/blisters in the BP disease area index (BPDAI) (*41*) were significantly higher in NC7-Col4(+) DPP4i-BP than in NC7-Col4(−) DPP4i-BP, with median scores of 24.0 and 8.0, respectively (*P* = 0.0458) (Fig. 6E and Table 1). However, other BPDAI scores, including total scores, were comparable between these groups (Fig. 6, F to H, and Table 1). Among the examined factors, age did not significantly differ between the two groups (Fig. 6I). The frequency of treatment without systemic corticosteroids was not significantly different between these groups (Table 1). To determine the utility of BP180 NC7-Col4–swapped ELISA in monitoring disease activity, we plotted BPDAI scores along with titers from full-length, NC16A, and NC7-Col4–swapped ELISAs in four cases throughout the disease course (fig. S5). In all cases, the titers of NC7-Col4–swapped ELISA were correlated with the disease activity of BP, although case 51 lacked BPDAI scores for the initial disease stage. Cases 3 and 51 showed epitope spreading, in which anti-BP180 NC16A autoantibodies emerged during the disease course. In case 3, the titers of anti-BP180 NC7-Col4 autoantibodies were elevated before the disease relapse. These data indicate that the measurement of anti-BP180 NC7-Col4 autoantibodies is more useful for the diagnosis and disease monitoring of DPP4i-BP than for the assessment of the initial disease severity and prognosis.

### Subclassification of NC7-Col4(+) DPP4i-BP based on anti-BP180 NC16A autoantibody status

As the presence of anti-BP180 NC16A and NC7-Col4 autoantibodies was not correlated (Fig. 6B), we further subclassified NC7-Col4(+) DPP4i-BP into two groups based on the presence of anti-BP180 NC16A autoantibodies: NC7-Col4(+) NC16A(+) DPP4i-BP and NC7-Col4(+) NC16A(−) DPP4i-BP. There were no significant differences in age or sex between these groups (fig. S6 and table S7). In addition, the index values and positive ratios of full-length BP180 ELISA, NC7-Col4–swapped ELISA, and BP230 ELISA did not significantly differ between these groups (fig. S6 and table S7). Of note, NC7-Col4(+) NC16A(+) DPP4i-BP exhibited significantly higher disease activity as assessed by BPDAI scores. Compared to the NC7-Col4(+) NC16A(−) group, they had higher median scores for the following: erosions/blisters (27 versus 9, *P* = 0.0017), urticaria/erythema (8 versus 0, *P* = 0.0096), and total BPDAI score (37 versus 13, *P* = 0.0001) (fig. S6 and table S7). In addition, NC7-Col4(+) NC16A(−) DPP4i-BP was more likely to achieve a complete response without systemic corticosteroids compared to NC7-Col4(+) NC16A(+) DPP4i-BP (71.4% versus 36.0%, *P* = 0.0484) (table S7). These findings indicate that the combined evaluation of anti-BP180 NC7-Col4 and NC16A autoantibodies is beneficial for assessing disease severity and prognosis, both of which were not predicted by measuring anti-BP180 NC7-Col4 autoantibodies only.

### Detection of anti-BP180 NC7-Col4 autoantibodies in DPP4i-treated patients with DM without BP development

**Fig. 7.**
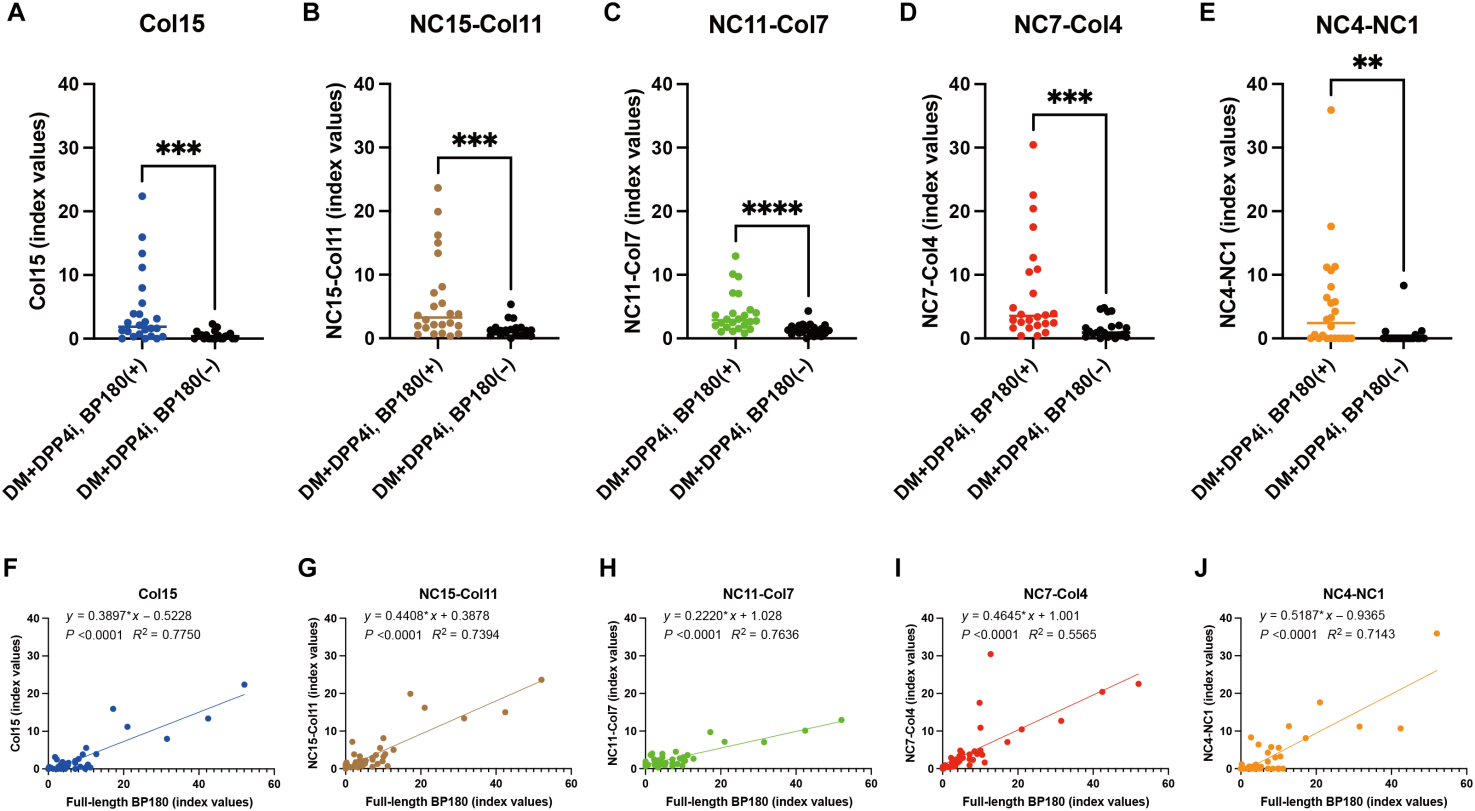
Comparison of domain-swapped ELISA index values in patients with DM + DPP4i who were positive and negative for anti-BP180 autoantibodies. (**A** to **E**) Comparison of index values for five domain-swapped BP180 ELISA index values, Col15 (A), NC15-Col11 (B), NC11-Col7 (C), NC7-Col4 (D), and NC4-NC1 (E), between patients with DM + DPP4i who were positive or negative for full-length BP180 autoantibodies. (**F** to **J**) Simple linear regression analysis of the index values of the full-length BP180 ELISA and each domain-swapped ELISA among anti-BP180(+) patients with DM + DPP4i. 0.001 < **P < 0.01; 0.0001 < ***P < 0.0001; ****P < 0.0001.

Last, we investigated whether anti-BP180 NC7-Col4 autoantibodies were present in patients with DM taking DPP4i (DM + DPP4i) before the onset of DPP4i-BP. In our previous study, we found that 10.9% of patients with DM + DPP4i who do not have developed BP had anti-BP180 autoantibodies, as detected by full-length BP180 ELISA (*34*). To define which BP180 regions these autoantibodies recognize, we compared sera from anti-BP180(+) patients with DM + DPP4i identified in that study to anti-BP180(−) patients with DM + DPP4i (Fig. 7 and table S8). The index values of all domain-swapped ELISAs were significantly higher in the anti-BP180(+) group than in the anti-BP180(−) group (Fig. 7, A to E, table S4). Moreover, the index values of each domain-swapped ELISA correlated with those of full-length BP180 ELISA (Fig. 7, F to J), although two patients showed disproportionately high reactivity in the NC7-Col4–swapped ELISA relative to the full-length BP180 ELISA (Fig. 7I). These findings demonstrated that even in the absence of clinical BP, patients with DM + DPP4i can develop autoantibodies against diverse BP180 epitopes—including the NC7-Col4 region—highlighting a preclinical humoral response that may presage DPP4i-BP development.

## DISCUSSION

In this study, we developed collagen-to-collagen domain-swapped molecules for epitope mapping of DPP4i-BP autoantibodies. Using this domain-swapped method revealed that the epitopes of DPP4i-BP are located in the NC11 to Col4 region of BP180 and that the NC7-Col4 region might be the primary epitope in DPP4i-BP.

Identifying precise epitopes, rather than assessing reactivity to the entire BP180 molecule, provides crucial insights into disease-specific immune targeting. In the case of DPP4i-BP, the preferential recognition of the NC7-Col4 region—distinct from the NC16A domain typically recognized in conventional BP—suggests the involvement of an alternative immunological pathway (*42*). This may be related to changes in antigen processing, presentation by specific HLA class II alleles (*13*, *39*), or loss of tolerance induced by DPP4 inhibition (*29*, *43*). Such epitope-level resolution enables the dissection of how particular regions of the autoantigen become immunogenic under specific conditions, contributing to our understanding of early disease pathogenesis (*29*, *44*). Moreover, the presence of these antibodies in some patients before clinical onset supports the notion that these epitopes may be involved in the initiation of the disease (*45*, *46*). Therefore, epitope mapping offers not only diagnostic refinement but also mechanistic insight into autoimmune blistering disease development (*47*, *48*).

The domain-swapping strategy is valuable for epitope mapping and has been applied to other autoimmune bullous diseases, such as pemphigus vulgaris and pemphigus foliaceus (*49*–*52*). This method has several advantages over previous approaches for DPP4i-BP autoantibody detection. WB using plasmin-cleaved BP180 revealed that DPP4i-BP has distinct autoantibody profiles compared to BP (*33*), although this method is unsuitable for evaluating many samples simultaneously in clinical settings compared to ELISA. Salemme *et al.* (*53*) used BP180 E-1080 (amino acids 1080 to 1107) and E-1331 (amino acids 1331 to 1404) ELISA using DPP4i-BP sera. These fragments partially span the NC9-Col8 and Col3-NC3-Col2 regions of BP180, respectively. However, the reported positive rates for DPP4i-BP were both 48%, and the authors did not find a statistical difference in positive rates between BP and DPP4i-BP groups (*53*). On the other hand, our domain-swapped BP180 NC7-Col4 ELISA efficiently detected autoantibodies of DPP4i-BP, yielding a sensitivity of 96% and a specificity of 100% for distinguishing between non-BP and NC16A(−) DPP4i-BP sera. The index value of BP180 NC7-Col4 ELISA was significantly elevated in NC16A(−) DPP4i-BP compared to BP, indicating that DPP4i-BP has distinct autoantibody profiles compared to BP.

Our findings on anti-BP180 NC7-Col4 autoantibodies are consistent with the genetic background of DPP4i-BP, where HLA-DQA1*05:05 and HLA-DQB1*03:01 are common (*39*). In this study, we searched for HLA class II candidate peptide epitopes of BP180 with high affinity for HLA-DQA1*05:05 and HLA-DQB1*03:01 using the netMHCIIpan4.3 algorithm (*40*), and the peptide with the highest binding site was the Col5-NC5 boundary region. We also experimentally confirmed that epitopes containing the Col5-NC5 region could be the primary epitope of DPP4i-BP, and patients with DPP4i-BP who have HLA-DQA1*05:05 and HLA-DQB1*03:01 tend to have anti-BP180 NC7-Col4 autoantibodies rather than those who have other HLAs. Because HLA plays a crucial role in antigen processing and presentation (*54*–*56*), these data suggest that anti-BP180 NC7-Col4 autoantibodies could be produced by antigen processing and/or presentation through HLA-DQA1*05:05 and HLA-DQB1*-03:01. This pathway may explain why DPP4i-BP has a distinct autoantibody profile compared to BP and highlights the unrevealed pathomechanism of DPP4i-BP. While these findings support a mechanistic link between the HLA genotype and epitope specificity, they must be interpreted in the context of ethnic variation. Our limitation of HLA analysis is that it is done among the Japanese population, in which HLA-DQB1*03:01 is enriched in DPP4i-BP (*13*, *39*). In the European population, this association is less pronounced (*57*), and HLA-DQB1*03:01 is reported to be associated with conventional BP (*58*), suggesting that the pathomechanism of HLA-linked antigen presentation may differ among ethnic groups. We need to conduct HLA-epitope analysis with ethnic diversity to generalize our hypothesis. Although our in silico analysis did not predict the sequence of the NC16A domain as a strong binding peptide, several reports showed that HLA-DQB1*03:01 is predicted to bind the NC16A domain (*59*, *60*). This potential binding capacity of HLA-DQB1*03:01 might be related to epitope spreading from the NC7-Col4 region to the NC16A domain in patients with DPP4i-BP.

**Fig. 8.**
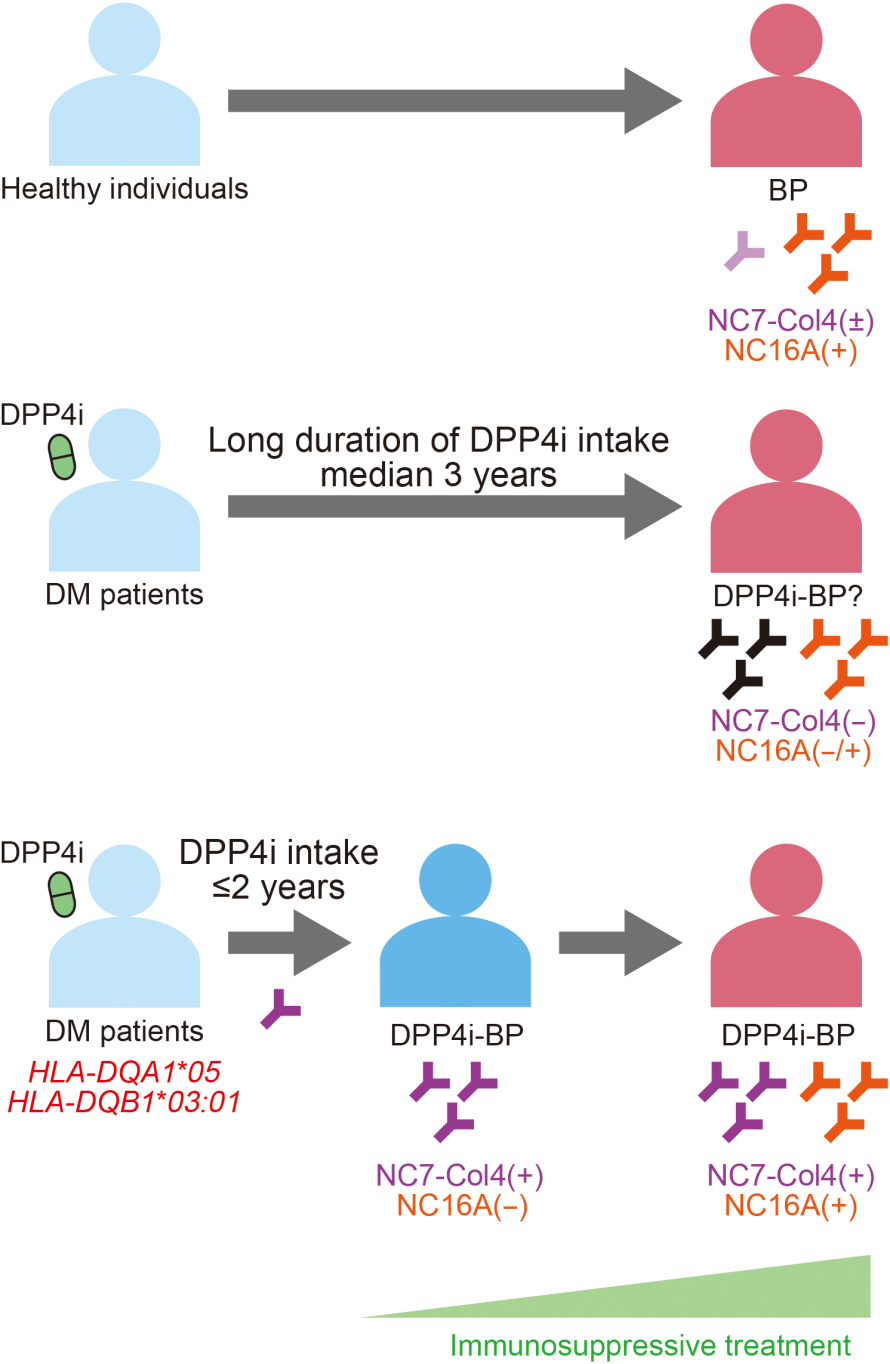
Schematic diagram showing the association of anti-BP180 autoantibodies with DPP4i-BP and BP. Patients with anti–NC7-Col4 autoantibodies had a significantly shorter DPP4i intake period before disease onset than those without anti–NC7-Col4 autoantibodies. In addition, epitope spreading to NC16A was associated with a requirement for more intensive treatment.

Detection of anti-BP180 NC7-Col4 autoantibodies provides important insights into the understanding of DPP4i-BP. Sitagliptin was the least-used DPP4i in NC7-Col4(+) DPP4i-BP, which is compatible with the recent findings that sitagliptin may not be associated with BP development (*29*, *61*). In addition, the onset of BP occurs more rapidly after DPP4i intake in NC7-Col4(+) DPP4i-BP than in NC7-Col4(−) DPP4i-BP (Fig. 8). These findings highlight the relevance of DPP4i in BP pathogenesis in NC7-Col4(+) DPP4i-BP but not in NC7-Col4(−) DPP4i-BP (Fig. 8). Combined with the findings on the DPP4i-BP–specific HLA affinity for the NC7-Col4 region of BP180, patients with DM and a genetic risk for producing anti-BP180 NC7-Col4 autoantibodies may develop BP via DPP4i intake. This also raises the possibility that NC7-Col4(−) DPP4i-BP might represent patients with BP coincidentally treated with DPP4i, but whose BP development is not actually associated with DPP4i intake. In this scenario, it might be plausible to continue DPP4i in patients with BP who do not have anti-BP180 NC7-Col4 autoantibodies.

Measuring anti-BP180 NC7-Col4 autoantibodies could be beneficial for monitoring disease activities. In our cases, the index values of anti-BP180 NC7-Col4 autoantibodies decreased in parallel with the recovery from the disease after treatment, while the index values of anti-BP180 NC7-Col4 autoantibodies increased before the relapse of BP. Anti-BP180 NC7-Col4 autoantibodies were present in patients with DM + DPP4i who do not have BP symptoms, which also raises the question of whether patients with DM + DPP4i who have anti-BP180 NC7-Col4 autoantibodies might have a higher risk of BP development than those without anti-BP180 NC7-Col4 autoantibodies. If this is the case, screening anti-BP180 NC7-Col4 autoantibodies could be a preventive approach against BP development in patients with DM + DPP4i. Further studies are warranted to answer these questions.

While our study focused on the epitope specificity of anti-BP180 NC7-Col4 autoantibodies, their pathogenicity remains unclear, especially in DM + DPP4i individuals without BP symptoms. One possible pathomechanism determining their pathogenicity might be immunoglobulin G (IgG) subclasses. It is known that the autoantibody class switch to IgG4 is related to the development of endemic pemphigus foliaceus (*62*–*64*). In contrast, IgG4 anti-BP180 autoantibodies can act protectively in BP by blocking IgG1 and IgG3 autoantibodies (*65*). Our previous study, which analyzed partially overlapping sera of our current study, demonstrated that the autoantibodies in DPP4i-BP were predominantly of the IgG1 subclass, while IgG4 autoantibodies were detected in only 38.9% of cases (*33*). From these studies, it might be possible that the autoantibody class switch to IgG1 may acquire pathogenicity in DPP4i-BP autoantibodies. Future studies examining the subclass characteristic of anti-BP180 NC7-Col4 autoantibodies are warranted to better define their role in disease pathogenesis.

Although measuring anti-BP180 NC7-Col4 autoantibodies is valuable for disease monitoring, the classification of patients with NC7-Col4(+) and NC7-Col4(−) DPP4i-BP did not contribute to the prediction of disease prognosis. Alternatively, patients with NC7-Col4(+) DPP4i-BP who have anti-BP180 NC16A autoantibodies required systemic corticosteroids more frequently to achieve a complete response than those who do not have anti-BP180 NC16A autoantibodies (Fig. 8). This might be explained by the fact that the continuous use of DPP4i leads to the exacerbation of BP symptoms and of epitope spreading that produces anti-BP180 NC16A autoantibodies resembling typical BP (*35*). These data highlight the clinical significance of the early diagnosis of DPP4i-BP before the emergence of anti-BP180 NC16A autoantibodies.

Beyond the diagnostic and mechanistic insights, our findings have important clinical implications. Identifying a highly specific autoantibody response targeting the NC7-Col4 region in DPP4i-BP suggests the potential use of this epitope as a biomarker for early detection or risk stratification in patients with diabetes treated with DPP4 inhibitors. Furthermore, epitope-specific profiling can meaningfully distinguish DPP4i-BP from conventional BP, thereby contributing to more accurate prognostic evaluation and guiding therapeutic decision-making. In addition, measuring anti–NC7-Col4 antibody levels provides a practical tool for assessing disease activity and monitoring the clinical course in DPP4i-BP.

In summary, this study successfully identified a distinct epitope profile in DPP4i-BP by applying a domain-swapped protein strategy that preserves the trimeric collagen conformation. We demonstrated that autoantibodies in DPP4i-BP preferentially target the NC7-Col4 region of BP180, in contrast to the NC16A-dominant reactivity in conventional BP. This epitope matches the predicted epitope of HLA-DQA1*05:05 and HLA-DQB1*03:01, which are predominant in Japanese patients with DPP4i-BP, suggesting the potential pathomechanism of the antigen presentation through specific HLAs in DPP4i-BP. Measuring anti–NC7-Col4 antibodies is beneficial for the early diagnosis of DPP4i-BP and the treatment decision.

## MATERIALS AND METHODS

### Serum samples

We retrospectively studied patients with BP and patients with suspected BP who had visited the Department of Dermatology, Hokkaido University Hospital, from 2013 to 2022, as well as patients with BP who had sent sera from other institutions and were diagnosed at our institution. We extracted data on age, gender, disease severity, treatment method, and prednisolone (PSL) dosage during treatment from the clinical records. Age and disease-severity values were presented at the time of the initial visit. Patients taking DPP4i at the onset of the disease were classified as DPP4i-BP. All patients with DM were diagnosed and treated with DPP4i at the Department of Diabetes and Endocrinology, Hokkaido Sapporo Kosei General Hospital, and had not developed BP at the time of serum collection.

### Patient characteristics

All patients with BP and DPP4i-BP were diagnosed on the basis of clinical, histopathological, and immunological findings as previously described (*1*). Serologic testing included indirect immunofluorescence using normal human skin and skin split with 1 M NaCl and/or ELISA or chemiluminescent enzyme immunoassay. On the basis of those results, serum samples were classified into three patient groups: patients without BP (non-BP), patients with BP, and patients with BP taking DPP4i at onset (DPP4i-BP). The patients without BP had inflammatory skin disease but did not satisfy the diagnostic criteria for BP (*1*), and autoantibodies to the basement membrane zone were not detected in either direct or indirect immunofluorescence.

The NC16A ELISA confirmed that the sera used in this study were negative for non-BP and NC16A(−) DPP4i-BP and positive for BP and (NC16A+) DPP4i-BP (fig. S1A). We also confirmed that non-BP sera were negative and DPP4i-BP sera were positive in the full-length BP180 ELISA (fig. S1B). All patients with BP had anti-BP180 NC16A autoantibodies, while all patients with DPP4i-BP had anti-BP180 autoantibodies targeting non-NC16A regions of BP180. Sera of several patients with DPP4i-BP and all patients with DM + DPP4i were used in our previous studies (*33*, *34*, *39*). Patient characteristics are summarized in Table 1.

### Ethics statement and consent

Approval for human studies was granted by the local ethics committee and the institutional review board of Hokkaido University (nos. 15-025 and 016-0061). Written informed consent was obtained from all participants.

### HLA typing

HLA typing was performed on 26 patients newly diagnosed with DPP4i-BP who were enrolled between 2015 and 2018. HLA genotyping of *HLA-A*, *HLA-B*, *HLA-C*, *HLA-DRB1*, *HLA-DQB1*, *HLA-DPB1*, and *HLA-DQA1* was performed as previously described (*13*) using a combination of polymerase chain reaction–based methods and sequencing, followed by haplotype phasing and allele assignment based on reference databases.

### Cell culture

Flp-In 293 cells (Invitrogen, Carlsbad, CA) were maintained in Dulbecco’s modified Eagle’s medium (Nacalai Tesque, Kyoto, Japan) supplemented with 10% fetal bovine serum (Invitrogen) and 1× antibiotic-antimycotic mixed solution (Nacalai Tesque) at 37°C in a humidified atmosphere with 5% CO_2_.

### Production of the COL13-BP180 domain-swapped protein expression plasmid

BP180 (NP_000485.3), a type II transmembrane collagen protein, consists of 1497 amino acids, with 15 Col domains in its extracellular domain interspersed with NC sequences (Fig. 1A) (*10*, *11*). C-terminal truncated forms of BP180—ΔNC3-NC1: Met^1^-Asp^1340^, ΔCol6-NC1: Met^1^-Arg^1174^, and ΔNC11-NC1: Met^1^-Pro^977^—were produced as previously reported (*66*). We also segmented the extracellular domain of BP180 into five regions: Col15 (Gly^567^-Ile^808^), NC15-Col11 (Val^809^-Ser^981^), NC11-Col7 (Glu^982^-Ser^1160^), NC7-Col4 (Tyr^1161^-Ser^1279^), and NC4-NC1 (Arg^1280^-Pro^1497^) (Fig. 2, A and B). In addition, we used Met^1^-Tyr^216^ (for domain-swapped Col15: Met^1^-Pro^120^) and Ala^700^-Lys^717^ regions of COL13 (NP_001123575.1) as a structural backbone for our constructs (Fig. 2C). To create the domain-swapped constructs, we first synthesized DNA sequences corresponding to the Met^1^-Tyr^216^ (for domain-swapped Col15: Met^1^-Pro^120^) and Ala^700^-Lys^717^ regions of COL13 (NM_001130103.1), incorporating *Nhe1* and *Apa1* restriction enzyme sites at the junctions and adding an N-terminal DDDDK tag (GenScript, Piscataway, NJ). Similarly, the DNA sequences for the BP180 extracellular domain fragments were synthesized, each flanked by *Nhe1* and *Apa1* restriction sites (GenScript). These BP180 segments were inserted into the COL13 backbone using the Nhe1 and Apa1 restriction enzymes. The full-length BP180 recombinant protein construct was prepared as previously described (*30*). The domain-swapped BP180 segments used in Fig. 4 were assembled using IVA (In Vivo Assembly) cloning (*67*), which fused the expression vector containing the COL13 backbone with the specific extracellular domain segment of BP180 (Fig. 4A).

### Establishment of cell lines stably expressing domain-swapped BP180 proteins

Plasmids were cotransfected with pOG44 (Invitrogen) into Flp-In 293 cells by Lipofectamine 2000 (Invitrogen). Transfected cells were selected under hygromycin B (200 μg/ml; Invitrogen) with Dulbecco’s modified Eagle’s medium containing 10% fetal bovine serum. After 48 hours posttransfection, hygromycin was added, and the culture was continued for 10 to 14 days. Expression of the recombinant proteins was confirmed by WB. Flp-In 293 cells stably expressing a domain-swapped BP180 recombinant protein were established as previously described (*30*).

### Purification of full-length BP180 and domain-swapped BP180 proteins

Full-length BP180 recombinant protein was produced as previously described (*30*). Flp-In 293 cells stably expressing targeted recombinant proteins at full confluency were lysed with cell lysis buffer [1% (v/v) nonidet P-40 (Nacalai Tesque), 25 mM tris-HCl (pH 7.4), 100 mM NaCl, 10 mM EDTA, and protease inhibitor cocktail (P8340, Sigma-Aldrich, Darmstadt, Germany)]. The whole-cell lysate was centrifuged at 13,000 rpm for 20 min at 4°C. The supernatant was immunoprecipitated with Anti-DDDDK-tag mAb-Magnetic Beads (M185-9, MBL, Tokyo, Japan), and then purified proteins were eluted with the DDDDK peptide (Sigma-Aldrich).

### SDS–polyacrylamide gel electrophoresis, Coomassie Brilliant Blue staining, and WB

Protein samples were denatured for 5 min at 95°C in 5× loading buffer (0.25 M tris-HCl, 8% SDS, 30% glycerol, 0.02% bromophenol blue, and 0.3 M β-mercaptoethanol; pH 6.8). Samples were run on a 12% SDS–polyacrylamide gel electrophoresis gel, followed by Coomassie Brilliant Blue staining. For WB, proteins were electrophoretically transferred onto nitrocellulose membranes (Bio-Rad). After the transfer, the membranes were blocked with 2% skim milk, followed by incubation with mouse anti-DDDDK IgG (F1804, RRID: AB_262044, Sigma-Aldrich) in a dilution of 1:1000 for 2 hours at room temperature. Patient sera were diluted 1:200 and incubated for 16 hours at 4°C. As a secondary antibody, the horseradish peroxidase (HRP)–conjugated anti-mouse antibody (115-036-006, RRID: AB_2338519, Jackson ImmunoResearch, West Grove, PA) or anti-human antibody (P0214, RRID: AB_2893418, Agilent Technologies, Santa Clara, CA) diluted 1:5000 in 2% skim milk blocking buffer was used. The membrane was incubated for 1 hour at room temperature. Protein bands were visualized via the Clarity Western ECL Substrate (Bio-Rad, Hercules, CA) and the LAS-4000 mini-Imager (Fujifilm, Tokyo, Japan).

### ELISA

For ELISA, flat-bottom 96-well plates were coated with the target recombinant proteins and incubated overnight at 4°C. The plates were washed and subsequently blocked using a blocking reagent for ELISA (Roche, Basel, Switzerland). Patient sera were prepared at a dilution of 1:100 and then added to the wells, incubating for 1 hour at room temperature. The positive control of the full-length BP180 ELISA comprised pooled sera from BP reacting with the NC16A domain of COL17, while the positive control of the domain-swapped ELISAs was mouse anti-DDDDK IgG (F1804, RRID: AB_262044, Sigma-Aldrich) in a dilution of 1:2,000. The negative controls were sera from healthy individuals. After washing, 1:5000 diluted HRP-conjugated mouse anti-human IgG antibody (P0214, RRID: AB_2893418, Agilent Technologies, Santa Clara, CA) or 1:10,000 diluted HRP-conjugated anti-mouse antibody (115-036-006, RRID: AB_2338519, Jackson ImmunoResearch, West Grove, PA) was added, and the plates were incubated for 1 hour at room temperature. Last, 3,3′,5,5′-tetramethylbenzidine was added as a substrate, and the absorbance was measured at 450 nm with the correlation wavelength set to 620 nm using a microplate reader (TECAN Austria GmbH, Grödig, Austria). BP180 NC16A ELISA was performed following the manufacturer’s instructions (MBL). The ELISA index value was calculated using the following formula

$$\begin{array}{c} \text{Index}\\ =\frac{\left({\text{OD}}_{450}\;\text{of tested serum}-{\text{OD}}_{450}\;\text{of negative control}\right)}{\left({\text{OD}}_{450}\;\text{of positive control}-{\text{OD}}_{450}\;\text{of negative control}\right)}\\ \times 100 \end{array}$$

### HLA class II peptide epitope prediction

For the prediction of HLA class II peptide epitopes of DPP4i-BP, we used the full-length BP180 protein as a reference. We focused on the alleles DQA1*05:05 and HLA-DQB1*03:01, notably prevalent in patients with DPP4i-BP in the Japanese population (*13*, *39*). To evaluate the binding affinity of the 15-nucleotide oligomer peptides, derived from the entire sequence of BP180, to HLA class II molecules, we used NetMHCIIpan-4.3 software (*40*), the whole data of which are available in the Supplementary Materials. We established a criterion for identifying potential epitopes: Only those peptides with a prediction score % rank of less than 2% were classified as strong binders. Although we entered the DQA1*05:05 and HLA-DQB1*03:01 pair as the input allele combination, the output was based on the closest available allele pair in the NetMHCIIpan 4.3 training set, DQA1*05:01 and HLA-DQB1*03:01, with a reported distance to training data of 0.000.

### Statistical analysis

*P* values were determined using the Mann-Whitney *U* test or Kruskal-Wallis test, followed by Dunn’s multiple comparisons test. *P* values < 0.05 were considered statistically significant. All statistical analyses were performed with GraphPad Prism 9.3 (GraphPad Software, San Diego, CA).

## References

[1] E. Schmidt, D. Zillikens, Pemphigoid diseases. Lancet 381, 320–332 (2013). 23237497 10.1016/S0140-6736(12)61140-4

[2] I. S. Bağcı, O. N. Horváth, T. Ruzicka, M. Sárdy, Bullous pemphigoid. Autoimmun. Rev. 16, 445–455 (2017). 28286109 10.1016/j.autrev.2017.03.010

[3] S. Egami, J. Yamagami, M. Amagai, Autoimmune bullous skin diseases, pemphigus and pemphigoid. J. Allergy Clin. Immunol. 145, 1031–1047 (2020). 32272980 10.1016/j.jaci.2020.02.013

[4] H. Ujiie, J. Yamagami, H. Takahashi, K. Izumi, H. Iwata, G. Wang, D. Sawamura, M. Amagai, D. Zillikens, The pathogeneses of pemphigus and pemphigoid diseases. J. Dermatol. Sci. 104, 154–163 (2021). 34916040 10.1016/j.jdermsci.2021.11.003

[5] K. Kridin, R. J. Ludwig, The growing incidence of bullous pemphigoid: Overview and potential explanations. Front. Med. (Lausanne) 5, 220 (2018). 30177969 10.3389/fmed.2018.00220 PMC6109638

[6] A. Ishiko, H. Shimizu, A. Kikuchi, T. Ebihara, T. Hashimoto, T. Nishikawa, Human autoantibodies against the 230-kD bullous pemphigoid antigen (BPAG1) bind only to the intracellular domain of the hemidesmosome, whereas those against the 180-kD bullous pemphigoid antigen (BPAG2) bind along the plasma membrane of the hemidesmosome in normal human and swine skin. J. Clin. Invest. 91, 1608–1615 (1993). 7682575 10.1172/JCI116368 PMC288138

[7] T. Masunaga, H. Shimizu, C. Yee, L. Borradori, Z. Lazarova, T. Nishikawa, K. B. Yancey, The extracellular domain of BPAG2 localizes to anchoring filaments and its carboxyl terminus extends to the lamina densa of normal human epidermal basement membrane. J. Invest. Dermatol. 109, 200–206 (1997). 9242508 10.1111/1523-1747.ep12319337

[8] H. Shimizu, New insights into the immunoultrastructural organization of cutaneous basement membrane zone molecules. Exp. Dermatol. 7, 303–313 (1998). 9858132 10.1111/j.1600-0625.1998.tb00329.x

[9] J. R. Stanley, Pemphigus and pemphigoid as paradigms of organ-specific, autoantibody-mediated diseases. J. Clin. Invest. 83, 1443–1448 (1989). 2651476 10.1172/JCI114036 PMC303845

[10] G. J. Giudice, D. J. Emery, L. A. Diaz, Cloning and primary structural analysis of the bullous pemphigoid autoantigen BP180. J. Invest. Dermatol. 99, 243–250 (1992). 1324962 10.1111/1523-1747.ep12616580

[11] B. Gatalica, L. Pulkkinen, K. Li, K. Kuokkanen, M. Ryynänen, J. A. McGrath, J. Uitto, Cloning of the human type XVII collagen gene (COL17A1), and detection of novel mutations in generalized atrophic benign epidermolysis bullosa. Am. J. Hum. Genet. 60, 352–365 (1997). 9012408 PMC1712405

[12] K. Nakama, H. Koga, N. Ishii, C. Ohata, T. Hashimoto, T. Nakama, Clinical and immunological profiles of 14 patients with bullous pemphigoid without IgG autoantibodies to the BP180 NC16A domain. JAMA Dermatol. 154, 347–350 (2018). 29299596 10.1001/jamadermatol.2017.5465 PMC5885813

[13] H. Ujiie, K. Muramatsu, T. Mushiroda, T. Ozeki, H. Miyoshi, H. Iwata, A. Nakamura, H. Nomoto, K. Y. Cho, N. Sato, M. Nishimura, T. Ito, K. Izumi, W. Nishie, H. Shimizu, HLA-DQB1*03:01 as a biomarker for genetic susceptibility to bullous pemphigoid induced by DPP-4 inhibitors. J. Invest. Dermatol. 138, 1201–1204 (2018). 29203362 10.1016/j.jid.2017.11.023

[14] Y. Tsuji-Abe, M. Akiyama, Y. Yamanaka, T. Kikuchi, K. C. Sato-Matsumura, H. Shimizu, Correlation of clinical severity and ELISA indices for the NC16A domain of BP180 measured using BP180 ELISA kit in bullous pemphigoid. J. Dermatol. Sci. 37, 145–149 (2005). 15734283 10.1016/j.jdermsci.2004.10.007

[15] Y. Mai, K. Izumi, K. Sawada, E. Akasaka, S. Mai, D. Sawamura, K. Ihara, S. Nakaji, W. Nishie, A 1,035-subject study suggesting a history of bone fracture as a possible factor associated with the development of anti-BP180 autoantibodies. J. Invest. Dermatol. 142, 984–987.e3 (2022). 34883045 10.1016/j.jid.2021.11.028

[16] Y. Mai, K. Izumi, S. Mai, W. Nishie, H. Ujiie, Detection of a natural antibody targeting the shed ectodomain of BP180 in mice. J. Dermatol. Sci. 112, 15–22 (2023). 37550175 10.1016/j.jdermsci.2023.07.009

[17] N. Oyama, J. F. Setterfield, A. M. Powell, Y. Sakuma-Oyama, S. Albert, B. S. Bhogal, R. W. Vaughan, F. Kaneko, S. J. Challacombe, M. M. Black, Bullous pemphigoid antigen II (BP180) and its soluble extracellular domains are major autoantigens in mucous membrane pemphigoid: The pathogenic relevance to HLA class II alleles and disease severity. Br. J. Dermatol. 154, 90–98 (2006). 16403100 10.1111/j.1365-2133.2005.06998.x

[18] A. Yasukochi, K. Teye, N. Ishii, T. Hashimoto, Clinical and immunological studies of 332 japanese patients tentatively diagnosed as anti-BP180-type mucous membrane pemphigoid: A novel BP180 C-terminal domain enzyme-linked immunosorbent assay. Acta Derm. Venereol. 96, 762–767 (2016). 26984589 10.2340/00015555-2407

[19] G. Du, S. Patzelt, N. van Beek, E. Schmidt, Mucous membrane pemphigoid. Autoimmun. Rev. 21, 103036 (2022). 34995762 10.1016/j.autrev.2022.103036

[20] C. Feliciani, G. Caldarola, A. Kneisel, E. Podstawa, M. Pfütze, W. Pfützner, M. Hertl, IgG autoantibody reactivity against bullous pemphigoid (BP) 180 and BP230 in elderly patients with pruritic dermatoses. Br. J. Dermatol. 161, 306–312 (2009). 19485996 10.1111/j.1365-2133.2009.09266.x

[21] T. Schmidt, C. Sitaru, K. Amber, M. Hertl, BP180- and BP230-specific IgG autoantibodies in pruritic disorders of the elderly: A preclinical stage of bullous pemphigoid? Br. J. Dermatol. 171, 212–219 (2014). 24601973 10.1111/bjd.12936

[22] Y. Mai, K. Izumi, S. Mai, H. Ujiie, The significance of preclinical anti-BP180 autoantibodies. Front. Immunol. 13, 963401 (2022). 36003369 10.3389/fimmu.2022.963401 PMC9393388

[23] J. Béné, G. Moulis, I. Bennani, M. Auffret, P. Coupe, S. Babai, D. Hillaire-Buys, J. Micallef, S. Gautier, French Association of Regional PharmacoVigilance Centres, Bullous pemphigoid and dipeptidyl peptidase IV inhibitors: A case–noncase study in the French Pharmacovigilance Database. Br. J. Dermatol. 175, 296–301 (2016). 27031194 10.1111/bjd.14601

[24] M. García, M. A. Aranburu, I. Palacios-Zabalza, U. Lertxundi, C. Aguirre, Dipeptidyl peptidase-IV inhibitors induced bullous pemphigoid: A case report and analysis of cases reported in the European pharmacovigilance database. J. Clin. Pharm. Ther. 41, 368–370 (2016). 27191539 10.1111/jcpt.12397

[25] M. Benzaquen, L. Borradori, P. Berbis, S. Cazzaniga, R. Valero, M.-A. Richard, L. Feldmeyer, Dipeptidyl peptidase IV inhibitors, a risk factor for bullous pemphigoid: Retrospective multicenter case-control study from France and Switzerland. J. Am. Acad. Dermatol. 78, 1090–1096 (2018). 29274348 10.1016/j.jaad.2017.12.038

[26] O. Varpuluoma, A.-K. Försti, J. Jokelainen, M. Turpeinen, M. Timonen, L. Huilaja, K. Tasanen, Vildagliptin significantly increases the risk of bullous pemphigoid: A Finnish nationwide registry study. J. Invest. Dermatol. 138, 1659–1661 (2018). 29427585 10.1016/j.jid.2018.01.027

[27] K. Kridin, R. Bergman, Association of bullous pemphigoid with dipeptidyl-peptidase 4 inhibitors in patients with diabetes: Estimating the risk of the new agents and characterizing the patients. JAMA Dermatol. 154, 1152–1158 (2018). 30090931 10.1001/jamadermatol.2018.2352 PMC6233738

[28] S. G. Lee, H. J. Lee, M. S. Yoon, D. H. Kim, Association of dipeptidyl peptidase 4 inhibitor use with risk of bullous pemphigoid in patients with diabetes. JAMA Dermatol. 155, 172–177 (2019). 30624566 10.1001/jamadermatol.2018.4556 PMC6439542

[29] K. Tasanen, O. Varpuluoma, W. Nishie, Dipeptidyl peptidase-4 inhibitor-associated bullous pemphigoid. Front. Immunol. 10, 1238 (2019). 31275298 10.3389/fimmu.2019.01238 PMC6593303

[30] K. Izumi, W. Nishie, Y. Mai, M. Wada, K. Natsuga, H. Ujiie, H. Iwata, J. Yamagami, H. Shimizu, Autoantibody profile differentiates between inflammatory and noninflammatory bullous pemphigoid. J. Invest. Dermatol. 136, 2201–2210 (2016). 27424319 10.1016/j.jid.2016.06.622

[31] H. Horikawa, Y. Kurihara, T. Funakoshi, N. Umegaki-Arao, H. Takahashi, A. Kubo, A. Tanikawa, N. Kodani, Y. Minami, S. Meguro, H. Itoh, K. Izumi, W. Nishie, H. Shimizu, M. Amagai, J. Yamagami, Unique clinical and serological features of bullous pemphigoid associated with dipeptidyl peptidase-4 inhibitors. Br. J. Dermatol. 178, 1462–1463 (2018). 29478242 10.1111/bjd.16479

[32] I. Ujiie, S. Katayama, Y. Mai, S. Mai, N. Yoshimoto, K. Muramatsu, H. Iwata, K. Izumi, H. Ujiie, Clinical characteristics and outcomes of dipeptidyl peptidase-4 inhibitor-associated bullous pemphigoid patients: A retrospective study. J. Am. Acad. Dermatol. 92, 561–564 (2024). 39442884 10.1016/j.jaad.2024.09.067

[33] Y. Mai, W. Nishie, K. Izumi, H. Shimizu, Preferential reactivity of dipeptidyl peptidase-IV inhibitor-associated bullous pemphigoid autoantibodies to the processed extracellular domains of BP180. Front. Immunol. 10, 1224 (2019). 31191560 10.3389/fimmu.2019.01224 PMC6549357

[34] K. Izumi, W. Nishie, M. Beniko, H. Shimizu, A cross-sectional study comparing the prevalence of bullous pemphigoid autoantibodies in 275 cases of type II diabetes mellitus treated with or without dipeptidyl peptidase-IV inhibitors. Front. Immunol. 10, 1439 (2019). 31297116 10.3389/fimmu.2019.01439 PMC6607930

[35] Y. Mai, W. Nishie, K. Izumi, N. Yoshimoto, Y. Morita, M. Watanabe, E. Toyonaga, H. Ujiie, H. Iwata, Y. Fujita, T. Nomura, K. C. Sato-Matsumura, S. Shimizu, H. Shimizu, Detection of anti-BP180 NC16A autoantibodies after the onset of dipeptidyl peptidase-IV inhibitor-associated bullous pemphigoid: A report of three patients. Br. J. Dermatol. 179, 790–791 (2018). 29624639 10.1111/bjd.16656

[36] H. Takama, M. Yoshida, K. Izumi, T. Yanagishita, J. Muto, Y. Ohshima, W. Nishie, H. Shimizu, M. Akiyama, D. Watanabe, Dipeptidyl peptidase-4 inhibitor-associated bullous pemphigoid: Recurrence with epitope spreading. Acta Dermato. Venereol. 98, 983–984 (2018). 10.2340/00015555-3010 30085319

[37] Y. Futei, M. Amagai, M. Sekiguchi, K. Nishifuji, Y. Fujii, T. Nishikawa, Use of domain-swapped molecules for conformational epitope mapping of desmoglein 3 in pemphigus vulgaris. J. Invest. Dermatol. 115, 829–834 (2000). 11069620 10.1046/j.1523-1747.2000.00137.x

[38] A. Snellman, A. Tuomisto, A. Koski, A. Latvanlehto, T. Pihlajaniemi, The role of disulfide bonds and α-helical coiled-coils in the biosynthesis of type XIII collagen and other collagenous transmembrane proteins. J. Biol. Chem. 282, 14898–14905 (2007). 17344215 10.1074/jbc.M609605200

[39] T. Ozeki, K. Muramatsu, N. Yoshimoto, I. Ujiie, K. Izumi, H. Iwata, T. Mushiroda, H. Ujiie, Association of genetic variants of HLA-DQA1 with bullous pemphigoid induced by dipeptidyl peptidase-4 inhibitors. J. Invest. Dermatol. 143, 2219–2225.e5 (2023). 37156394 10.1016/j.jid.2023.04.017

[40] J. B. Nilsson, S. Kaabinejadian, H. Yari, M. G. D. Kester, P. van Balen, W. H. Hildebrand, M. Nielsen, Accurate prediction of HLA class II antigen presentation across all loci using tailored data acquisition and refined machine learning. Sci. Adv. 9, eadj6367 (2023). 38000035 10.1126/sciadv.adj6367 PMC10672173

[41] D. F. Murrell, B. S. Daniel, P. Joly, L. Borradori, M. Amagai, T. Hashimoto, F. Caux, B. Marinovic, A. A. Sinha, M. Hertl, P. Bernard, D. Sirois, G. Cianchini, J. A. Fairley, M. F. Jonkman, A. G. Pandya, D. Rubenstein, D. Zillikens, A. S. Payne, D. Woodley, G. Zambruno, V. Aoki, C. Pincelli, L. Diaz, R. P. Hall, M. Meurer, J. M. Mascaro, E. Schmidt, H. Shimizu, J. Zone, R. Swerlick, D. Mimouni, D. Culton, J. Lipozencic, B. Bince, S. A. Grando, J.-C. Bystryn, V. P. Werth, Definitions and outcome measures for bullous pemphigoid: Recommendations by an international panel of experts. J. Am. Acad. Dermatol. 66, 479–485 (2012). 22056920 10.1016/j.jaad.2011.06.032 PMC3883429

[42] E. Schmidt, D. Zillikens, Modern diagnosis of autoimmune blistering skin diseases. Autoimmun. Rev. 10, 84–89 (2010). 20713186 10.1016/j.autrev.2010.08.007

[43] W. Nishie, K. Tasanen, Gliptin-associated bullous pemphigoid: A valuable model of the mechanism of breakdown of immune tolerance against BP180. J. Invest. Dermatol. 139, 755–756 (2019). 30904079 10.1016/j.jid.2018.11.025

[44] G. D. Zenzo, S. Thoma-Uszynski, V. Calabresi, L. Fontao, S. C. Hofmann, J.-P. Lacour, F. Sera, L. Bruckner-Tuderman, G. Zambruno, L. Borradori, M. Hertl, Demonstration of epitope-spreading phenomena in bullous pemphigoid: Results of a prospective multicenter study. J. Invest. Dermatol. 131, 2271–2280 (2011). 21697892 10.1038/jid.2011.180

[45] E. Raneses, M. M. Simpson, A. Rosenberg, M. Coffman, J. Meyerle, A retrospective review of autoimmune bullous disease antibody positivity before clinical symptoms. J. Am. Acad. Dermatol. 86, 237–239 (2022). 33549648 10.1016/j.jaad.2021.02.003

[46] W. Prüßmann, J. Prüßmann, H. Koga, A. Recke, H. Iwata, D. Juhl, S. Görg, R. Henschler, T. Hashimoto, E. Schmidt, D. Zillikens, S. M. Ibrahim, R. J. Ludwig, Prevalence of pemphigus and pemphigoid autoantibodies in the general population. Orphanet J. Rare Dis. 10, 63 (2015). 25971981 10.1186/s13023-015-0278-x PMC4436865

[47] G. D. Zenzo, F. Grosso, M. Terracina, F. Mariotti, A. Mastrogiacomo, F. Sera, G. Zambruno, O. D. Pità, K. Owaribe, L. Borradori, Characterization of the anti-BP180 autoantibody reactivity profile and epitope mapping in bullous pemphigoid patients. J. Invest. Dermatol. 122, 103–110 (2004). 14962097 10.1046/j.0022-202X.2003.22126.x

[48] M. Wada, W. Nishie, H. Ujiie, K. Izumi, H. Iwata, K. Natsuga, H. Nakamura, Y. Kitagawa, H. Shimizu, Epitope-dependent pathogenicity of antibodies targeting a major bullous pemphigoid autoantigen collagen XVII/BP180. J. Invest. Dermatol. 136, 938–946 (2016). 26827765 10.1016/j.jid.2015.11.030

[49] B. Ohyama, K. Nishifuji, P. T. Chan, A. Kawaguchi, T. Yamashita, N. Ishii, T. Hamada, T. Dainichi, H. Koga, D. Tsuruta, M. Amagai, T. Hashimoto, Epitope spreading is rarely found in pemphigus vulgaris by large-scale longitudinal study using desmoglein 2–based swapped molecules. J. Invest. Dermatol. 132, 1158–1168 (2012). 22277941 10.1038/jid.2011.448

[50] P. T. Chan, B. Ohyama, K. Nishifuji, K. Yoshida, K. Ishii, T. Hashimoto, M. Amagai, Immune response towards the amino-terminus of desmoglein 1 prevails across different activity stages in nonendemic pemphigus foliaceus. Br. J. Dermatol. 162, 1242–1250 (2010). 20163417 10.1111/j.1365-2133.2010.09696.x

[51] A. Cho, A. L. Caldara, N. A. Ran, Z. Menne, R. C. Kauffman, M. Affer, A. Llovet, C. Norwood, A. Scanlan, G. Mantus, B. Bradley, S. Zimmer, T. Schmidt, M. Hertl, A. S. Payne, R. Feldman, A. P. Kowalczyk, J. Wrammert, Single-cell analysis suggests that ongoing affinity maturation drives the emergence of pemphigus vulgaris autoimmune disease. Cell Rep. 28, 909–922.e6 (2019). 31340153 10.1016/j.celrep.2019.06.066 PMC6684256

[52] H. Koga, K. Teye, Y. Otsuji, N. Ishii, T. Hashimoto, T. Nakama, Autoantibodies to DSC3 in pemphigus exclusively recognize calcium-dependent epitope in extracellular domain 2. J. Invest. Dermatol. 141, 2123–2131.e2 (2021). 33766509 10.1016/j.jid.2021.01.032

[53] A. Salemme, L. Fania, A. Scarabello, M. Caproni, A. V. Marzano, E. Cozzani, C. Feliciani, C. D. Simone, M. Papini, R. R. Satta, A. Parodi, F. Mariotti, S. Lechiancole, G. Genovese, F. Passarelli, F. Festa, B. Bellei, A. Provini, D. Sordi, S. Pallotta, D. Abeni, C. Mazzanti, B. Didona, G. Di Zenzo, Cutaneous Immunology Group of SIDeMaST, Gliptin-associated bullous pemphigoid shows peculiar features of anti-BP180 and -BP230 humoral response: results from a multicenter study. J. Am. Acad. Dermatol. 87, 56–63 (2022). 35240229 10.1016/j.jaad.2022.02.036

[54] J. S. Blum, P. A. Wearsch, P. Cresswell, Pathways of antigen processing. Annu. Rev. Immunol. 31, 443–473 (2013). 23298205 10.1146/annurev-immunol-032712-095910 PMC4026165

[55] J. Neefjes, M. L. M. Jongsma, P. Paul, O. Bakke, Towards a systems understanding of MHC class I and MHC class II antigen presentation. Nat. Rev. Immunol. 11, 823–836 (2011). 22076556 10.1038/nri3084

[56] K. L. Rock, E. Reits, J. Neefjes, Present yourself! By MHC class I and MHC class II molecules. Trends Immunol. 37, 724–737 (2016). 27614798 10.1016/j.it.2016.08.010 PMC5159193

[57] O. Lindgren, O. Varpuluoma, J. Tuusa, J. Ilonen, L. Huilaja, N. Kokkonen, K. Tasanen, Gliptin-associated bullous pemphigoid and the expression of dipeptidyl peptidase-4/CD26 in bullous pemphigoid. Acta Dermato. Venereol. 99, 602–609 (2019). 10.2340/00015555-3166 30848289

[58] K. T. Amber, J. Zikry, M. Hertl, A multi-hit hypothesis of bullous pemphigoid and associated neurological disease: Is HLA-DQB1*03:01, a potential link between immune privileged antigen exposure and epitope spreading? HLA 89, 127–134 (2017). 28101965 10.1111/tan.12960

[59] L. R. Zakka, P. Reche, A. R. Ahmed, Role of MHC class II genes in the pathogenesis of pemphigoid. Autoimmun. Rev. 11, 40–47 (2011). 21782980 10.1016/j.autrev.2011.07.002

[60] L. R. Zakka, P. A. Reche, A. R. Ahmed, The molecular basis for the presence of two autoimmune diseases occurring simultaneously–Preliminary observations based on computer analysis. Autoimmunity 45, 253–263 (2012). 22053914 10.3109/08916934.2011.632454

[61] D. Shalmon, E. Bar-ilan, A. Peled, S. Geller, J. Bar, N. Schwartz, E. Sprecher, M. Pavlovsky, Identification of risk factors for gliptin-associated bullous pemphigoid among diabetic patients. Acta Derm. Venereol. 104, 26663 (2024). 38576104 10.2340/actadv.v104.26663 PMC11005169

[62] B. Rock, C. R. Martins, A. N. Theofilopoulos, R. S. Balderas, G. J. Anhalt, R. S. Labib, S. Futamura, E. A. Rivitti, L. A. Diaz, The pathogenic effect of IgG4 autoantibodies in endemic pemphigus foliaceus (Fogo Selvagem). N. Engl. J. Med. 320, 1463–1469 (1989). 2654636 10.1056/NEJM198906013202206

[63] S. J. P. Warren, L. A. Arteaga, L. A. Diaz, E. A. Rivitti, V. Aoki, G. Hans-Filho, B. F. Qaqish, M. S. Lin, G. J. Giudice, The role of subclass switching in the pathogenesis of endemic pemphigus foliaceus. J. Invest. Dermatol. 120, 1–5 (2003). 10.1046/j.1523-1747.2003.12017.x 12535205

[64] F. Evangelista, A. J. Roth, P. Prisayanh, B. R. Temple, N. Li, Y. Qian, D. A. Culton, Z. Liu, O. J. Harrison, J. Brasch, B. Honig, L. Shapiro, L. A. Diaz, Pathogenic IgG4 autoantibodies from endemic pemphigus foliaceus recognize a desmoglein-1 conformational epitope. J. Autoimmun. 89, 171–185 (2018). 29307589 10.1016/j.jaut.2017.12.017 PMC5902409

[65] Y. Zuo, F. Evangelista, D. Culton, A. Guilabert, L. Lin, N. Li, L. Diaz, Z. Liu, IgG4 autoantibodies are inhibitory in the autoimmune disease bullous pemphigoid. J. Autoimmun. 73, 111–119 (2016). 27377454 10.1016/j.jaut.2016.06.019 PMC5003671

[66] W. Nishie, D. Kiritsi, A. Nyström, S. C. Hofmann, L. Bruckner-Tuderman, Dynamic interactions of epidermal collagen XVII with the extracellular matrix laminin 332 as a major binding partner. Am. J. Pathology 179, 829–837 (2011). 10.1016/j.ajpath.2011.04.019 PMC3157233 21801871

[67] J. García-Nafría, J. F. Watson, I. H. Greger, IVA cloning: A single-tube universal cloning system exploiting bacterial in vivo assembly. Sci. Rep. 6, 27459 (2016). 27264908 10.1038/srep27459 PMC4893743

