# Supplementary material for: Identification of distinct epitopes in dipeptidyl peptidase-4 inhibitor–associated bullous pemphigoid
Source: Sci Adv. 2025 Aug 1;11(31):eadv9423. doi: 10.1126/sciadv.adv9423 (PMC12315971; doi:10.1126/sciadv.adv9423)
Supplement: Supplementary file 1 — Figs. S1 to S6 Tables S1 to S8 Legend for data S1 [file sciadv.adv9423_sm.pdf]

Science Advances
AAAS

Supplementary Materials for

Identification of distinct epitopes in dipeptidyl peptidase-4 inhibitor–associated bullous pemphigoid

Shoko Mai *et al.*

Corresponding author: Shoko Mai, shokomaiderma@gmail.com; Hideyuki Ujiie, h-ujiie@med.hokudai.ac.jp

Sci. Adv. **11**, eadv9423 (2025)
DOI: 10.1126/sciadv.adv9423

The PDF file includes:

Figs. S1 to S6
Tables S1 to S8
Legend for data S1

Other Supplementary Material for this manuscript includes the following:

Data S1

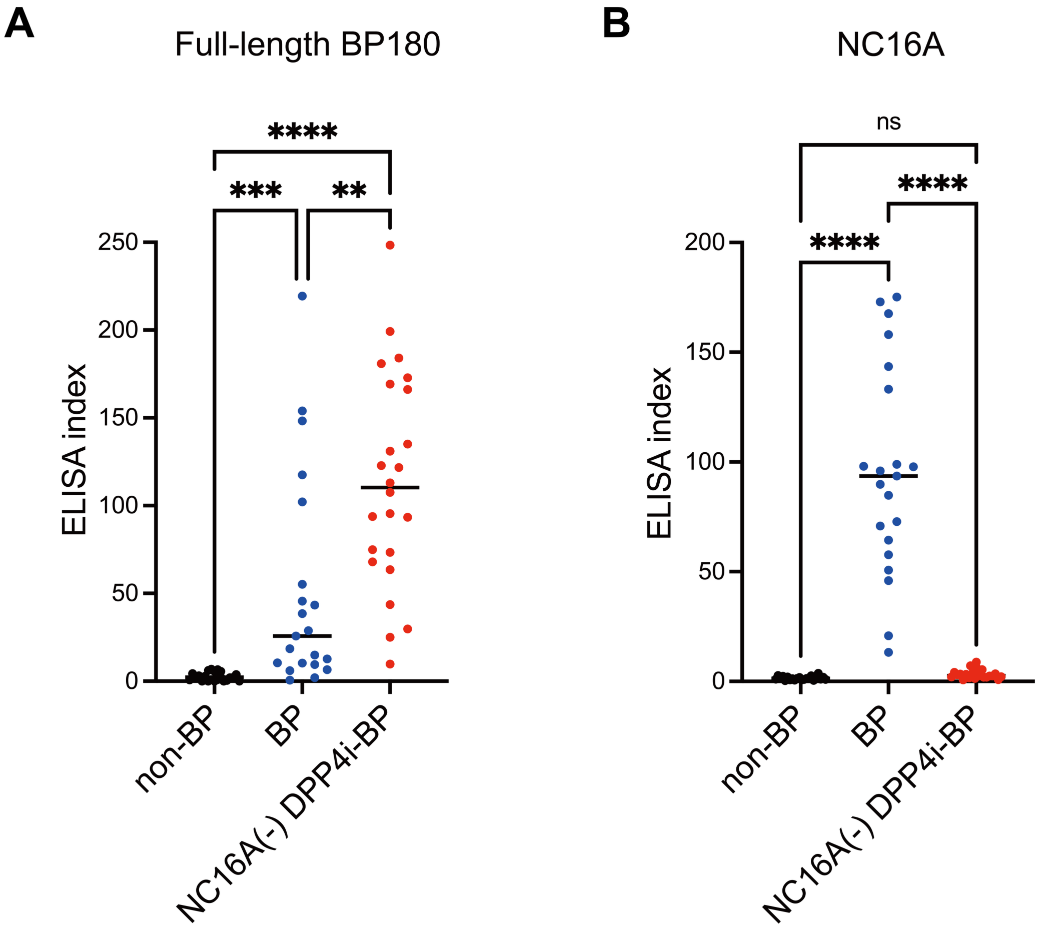

Fig. S1.

Serological confirmation of non-BP, BP, and DPP4i-BP (NC16A-) used in this study.
(**A**) Confirmation that the full-length BP180 ELISA was negative for non-BP sera and positive for NC16A(-) DPP4i-BP sera. (**B**) Similarly, confirmation that the sera used in this study were negative for non-BP and NC16A(-) DPP4i-BP, and positive for BP.

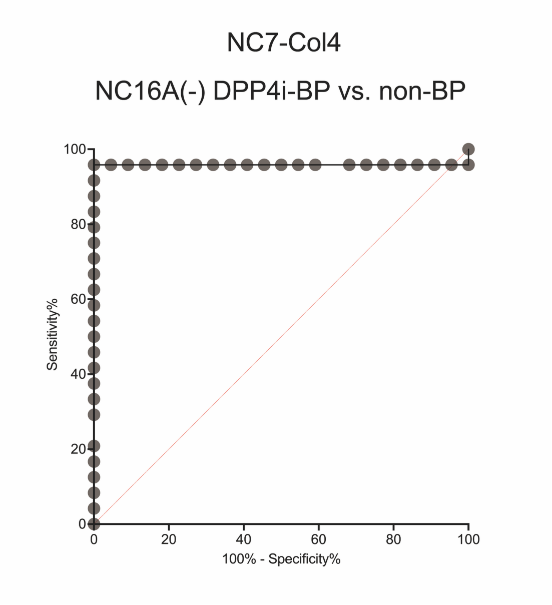

Fig. S2.

ROC curve of NC16A(-) DPP4i-BP vs. non-BP.

(**A**) We determined a cutoff value of 8.485, which gave the NC7-Col4 ELISA a sensitivity of 96% and specificity of 100% for NC16A(-) DPP4i-BP vs. non-BP.

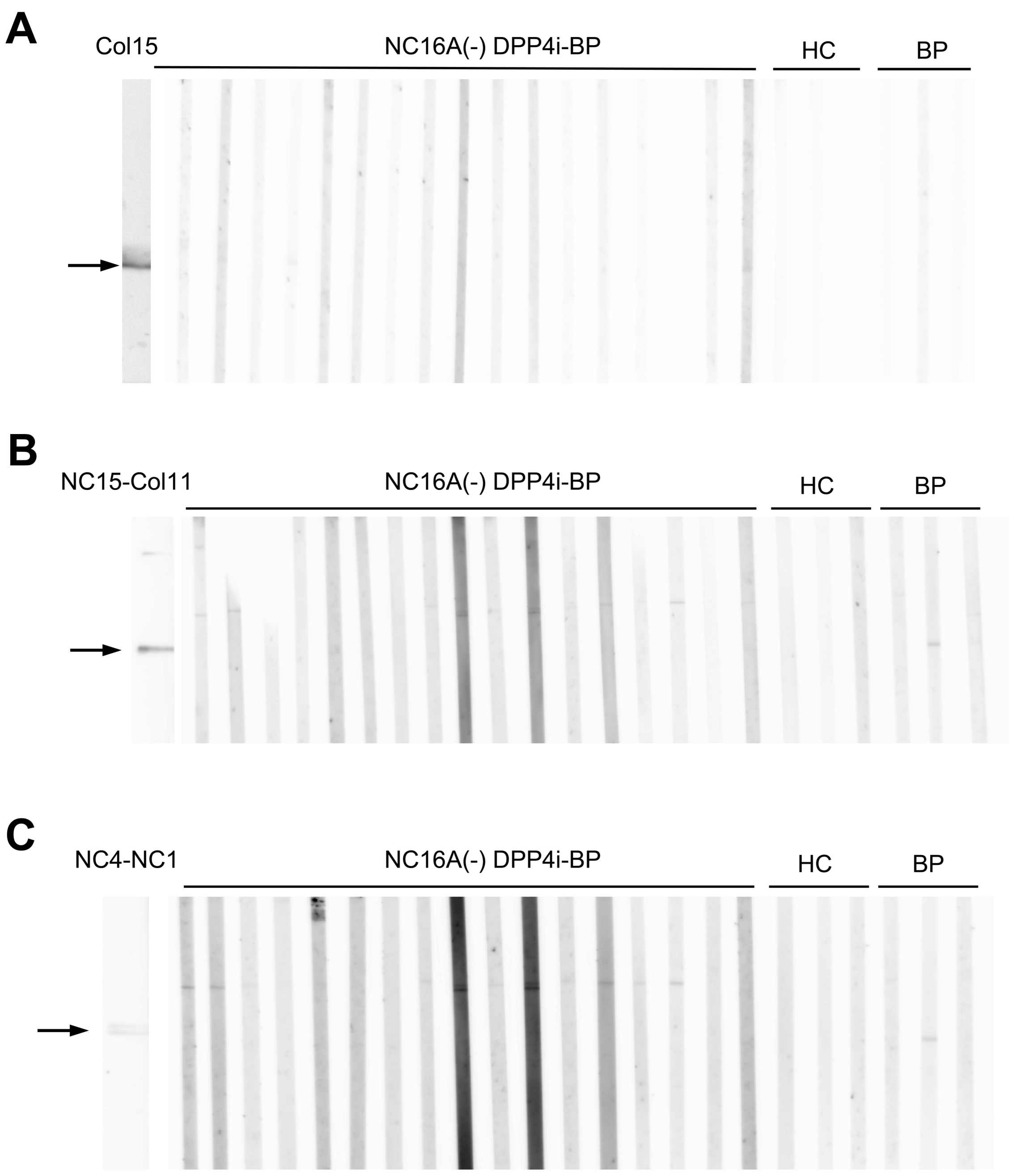

Fig. S3.

Western blotting with patient sera for Col15, NC15-Col11, and NC4-NC1 domain-swapped BP180 recombinant protein.

(**A-C**) Against Col15, NC15-Col11, and NC4-NC1, respectively. Neither NC16A(-) DPP4i-BP, BP, nor the negative control responded to any of these recombinant proteins.

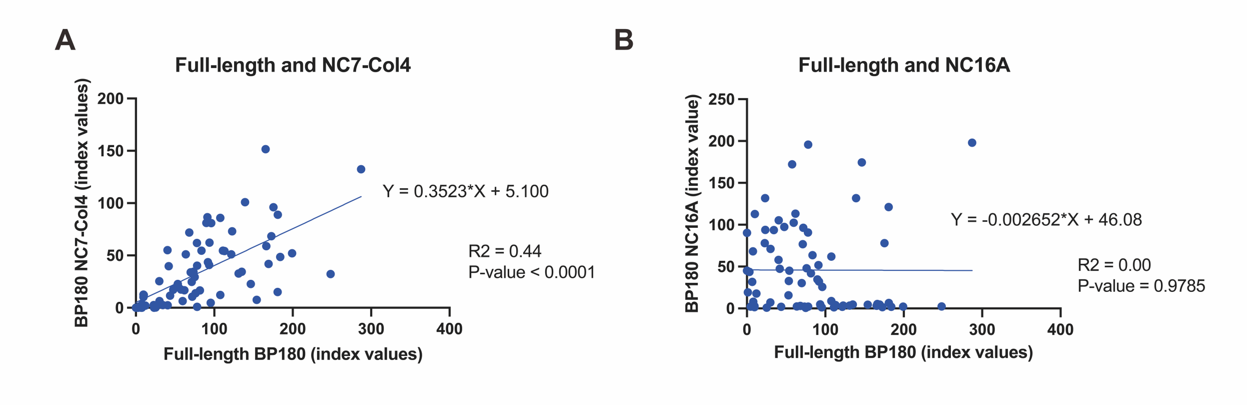

Fig. S4.

Simple linear regression analysis of the index values of the full-length BP180 ELISA and the NC7-Col4-swapped ELISA or NC16A ELISA.
(**A**) The index value of the full-length BP180 ELISA was significantly correlated with the index value of the BP180 NC7-Col4-swapped ELISA (*P*-value < 0.0001, y = 0.3523x + 5.100). (**B**) On the other hand, the index values of full-length BP180 ELISA and BP180 NC16A ELISA were not correlated (*P*-value = 0.9785).

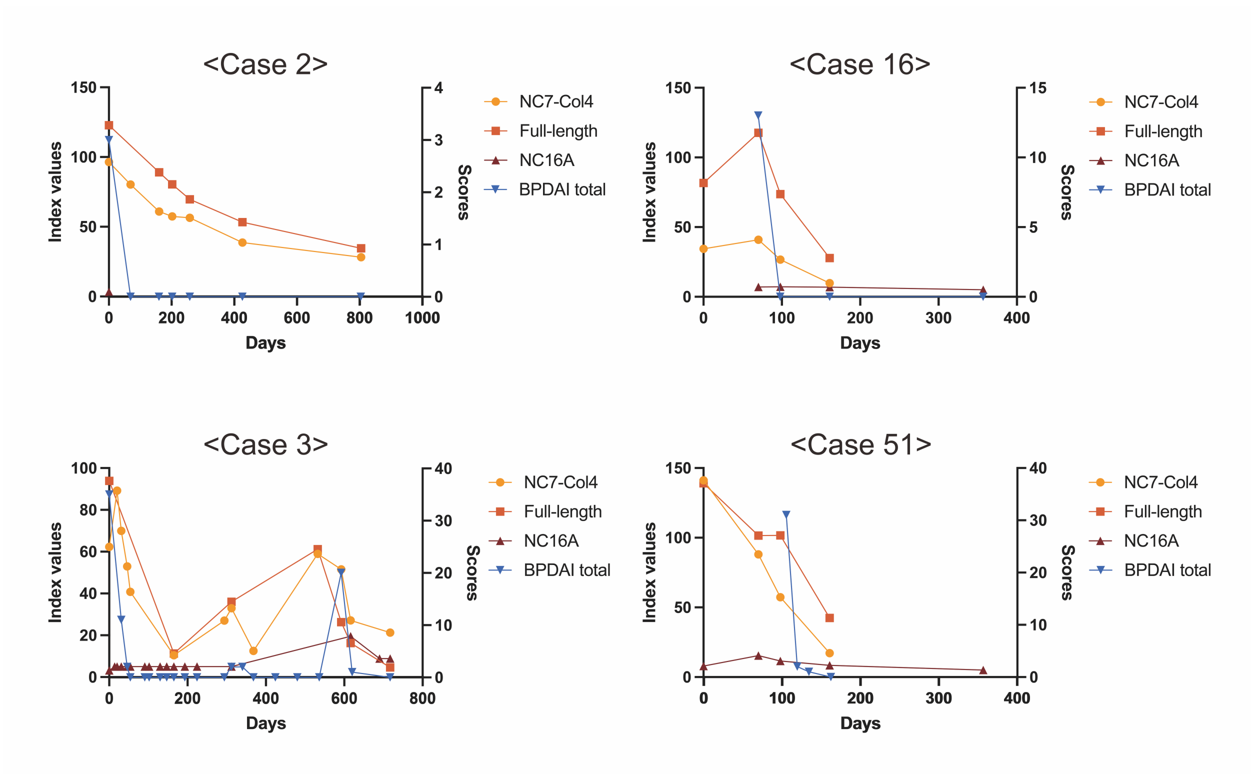

Fig. S5.

Time course of index values of NC7-Col4, full-length BP180, and NC16A ELISAs and BPDAI scores in four cases.

In Case 3 and Case 51, epitope spreading occurred during the disease, and anti-BP180 NC16A autoantibodies became positive. In all cases, there was a decrease in BPDAI score and in NC7-Col4 and full-length BP180 ELISA index values after the start of treatment. In Case 3, anti-BP180 NC7-Col4 autoantibodies were elevated before relapse.

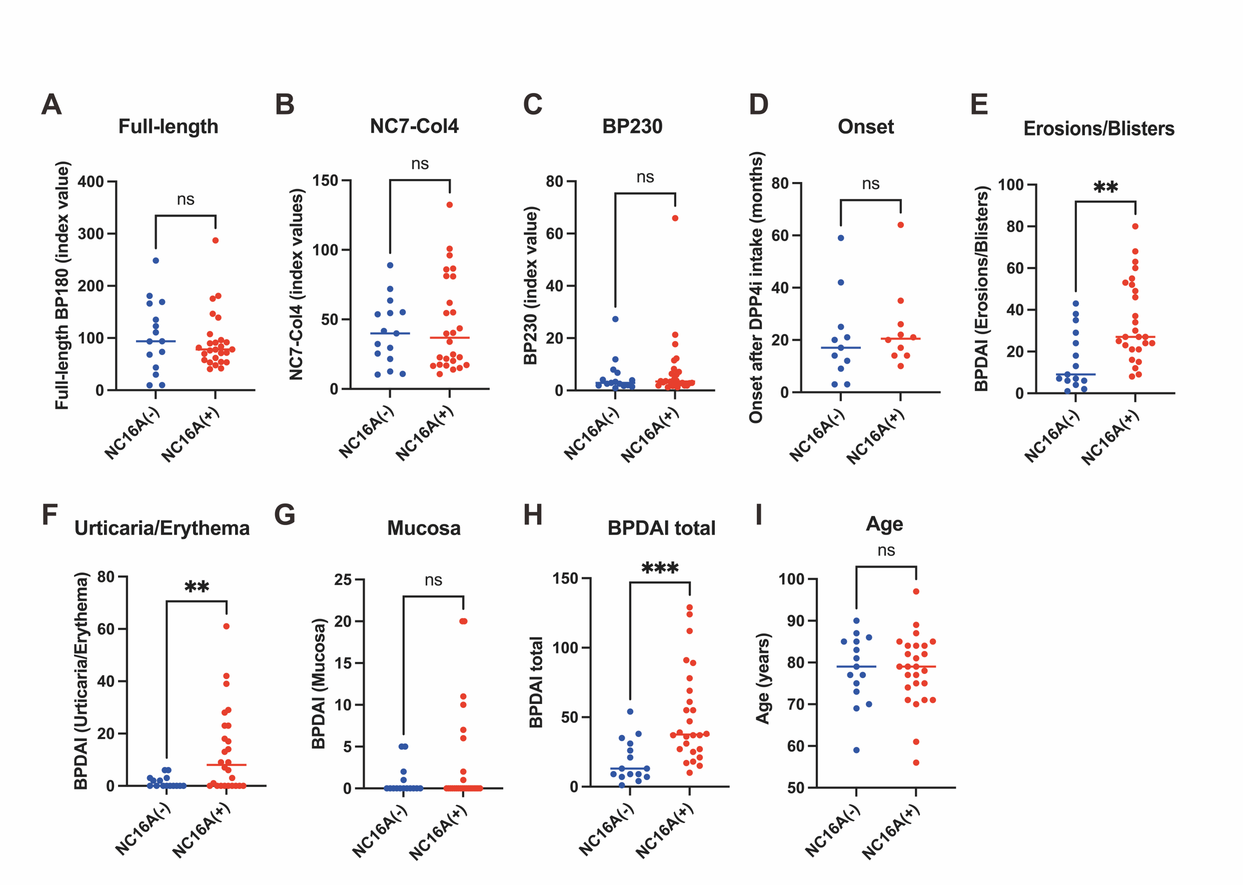

Fig. S6.

Subclassification of NC7-Col4(+) DPP4i-BP patients with and without anti-BP180 NC16A autoantibodies.

(**A-I**) Scatterplot comparing NC7-Col4(+) DPP4i-BP patients with and without NC16A autoantibodies for each factor. (**A, B, C, D, G, I**) Index values of full-length BP180 ELISA, BP180 NC7-Col4-swapped ELISA, BP230 ELISA, onset, mucosa BPDAI scores, and age did not significantly differ between these groups. (**E, F, H**) NC7-Col4(+) NC16A(+) DPP4i-BP showed higher scores than NC7-Col4(+) NC16A(-) DPP4i-BP for Blister/Erosion, Urticaria/Erythema, and total BPDAI scores.

Table S1. Demographic and clinical characteristics of NC16A(-) DPP4i-BP patients from Fig. 1.

	NC16A(-) DPP4i-BP (N=37)		
**Characteristics**	**Total (N=37)**	**For WB (N=22)**	**For ELISA (N=15)**
**Age, median (range), years**	76 (53-91)	76 (53-91)	76 (56-86)
**Female sex, n (%)**	11 (29.7)	7 (31.8)	4 (26.7)
**Full-length BP180**			
**Median (range), index value**	93.7 (8.2-248.4)	101.6 (8.2-248.4)	85.5 (15.8-135.4)
**Positive rate, n (%)**	37 (100)	22 (100)	15 (100)

Table S2. Demographic and clinical characteristics of non-BP, BP, NC16A(-) DPP4i-BP patients from Fig. 3.

Characteristics	Non-BP (N=22)	BP (N=21)	NC16A(-) DPP4i-BP (N=24)
**Age, median (range), years**	73 (56-90)	78 (43-94)	79 (53-91)
**Female sex, n (%)**	10 (45.5)	10 (47.6)	7 (29.2)
**Full-length BP180**			
**Median (range), index value**	2.3 (0.0-6.7)	25.8 (0.6-219.3)	110.3 (9.8-248.4)
**Positive rate, n (%)**	5 (22.7)	19 (90.5)	24 (100)
**BP180 NC16A**			
**Median (range), index value**	1.3 (0.4-3.7)	93.6 (13.3-175.2)	2.7 (0.7-8.9)
**Positive rate, n (%)**	0 (0)	21 (100)	0 (0)

Table S3. DPP4i use in NC16A(-) DPP4i-BP patients from Fig. 3.

DPP4i	n (%)
**Teneligliptin**	7 (33.3)
**Vildagliptin**	6 (28.6)
**Linagliptin**	5 (23.8)
**Sitagliptin**	3 (14.3)
**Alogliptin**	2 (9.5)
**Anagliptin**	2 (9.5)
**Omarigliptin**	1 (4.8)

Table S4. Details of the HLA-class II peptide epitopes of BP180 with high affinity for *HLA-DQA1*05:05* and *HLA-DQB1*03:01* using the netMHCIIpan4.3.

Rank	Position	Epitope sequence	Peptide core	%Rank
1	1228	PPGVSGALATYAAEN	VSGALATYA	0.02
	1227	GPPGVSGALATYAAE	VSGALATYA	0.04
	1229	PGVSGALATYAAENS	VSGALATYA	0.12
	1226	RGPPGVSGALATYAA	VSGALATYA	0.2
	1230	GVSGALATYAAENSD	VSGALATYA	1.19
	1225	PRGPPGVSGALATYA	VSGALATYA	1.94
2	248	TNAYSAGSVFGVPNN	YSAGSVFGV	0.06
	247	NTNAYSAGSVFGVPN	YSAGSVFGV	0.13
	249	NAYSAGSVFGVPNNM	YSAGSVFGV	0.26
	246	LNTNAYSAGSVFGVP	YSAGSVFGV	0.49
	250	AYSAGSVFGVPNNMA	YSAGSVFGV	1.94
3	1340	DIGPGGGYGAAAEGG	PGGGYGAAA	0.22
	1339	TDIGPGGGYGAAAEG	PGGGYGAAA	0.4
	1341	IGPGGGYGAAAEGGM	PGGGYGAAA	0.69
	1338	GTDIGPGGGYGAAAE	PGGGYGAAA	0.82
	1342	GPGGGYGAAAEGGMY	PGGGYGAAA	1.31
4	192	ETKIVTASSQSVSGT	IVTASSQSV	0.48
	191	VETKIVTASSQSVSG	IVTASSQSV	0.63
	190	TVETKIVTASSQSVS	IVTASSQSV	1.61
	193	TKIVTASSQSVSGTY	IVTASSQSV	1.84
5	367	SGKVFTASPASIAAT	FTASPASIA	1.11
	368	GKVFTASPASIAATS	FTASPASIA	1.27
	366	DSGKVFTASPASIAA	VFTASPASI	1.64
6	162	KRLLKGSRSASVSPT	LKGSRSASV	1.39
	161	VKRLLKGSRSASVSP	LKGSRSASV	1.83
7	106	RHAYEGSSSGNSSPE	YEGSSSGNS	1.91

Table S5. Domain-swapped ELISA in HLA-typed DPP4i-BP patients

Characteristics	DPP4i-BP (N=26)	HLA (+) (N=18)	HLA (-) (N=8)
**Age, median (range), years**	77 (49-97)	78 (59-90)	75 (49-97)
**Female sex, n (%)**	6 (23.1)	5 (27.8)	1 (12.5)
**DPP4i, n (%)**			
**Vildagliptin**	9 (34.6)	7 (38.9)	2 (25.0)
**Linagliptin**	7 (26.9)	4 (22.2)	3 (37.5)
**Sitagliptin**	4 (15.4)	1 (5.6)	3 (37.5)
**Teneligliptin**	4 (15.4)	3 (16.7)	1 (12.5)
**Alogliptin**	4 (15.4)	3 (16.7)	1 (12.5)
**Anagliptin**	1 (3.8)	1 (5.6)	0 (0.0)
**Full-length BP180**			
**Median (range), index value**	153.43 (6.19-287.1)	78.9 (6.19-287.1)	41.1 (7.6-135.1)
**Positive cases, n (%)**	26 (100)	18 (100)	8 (100)
**BP180 NC16A**			
**Median (range), index value**	33.7 (2.0-198.1)	31.46(2.0-198.1)	80.9 (4.78-131.8)
**Positive cases, n (%)**	17 (65.3)	10 (55.6)	7 (87.5)
**BP180 Col15, median (range), index value**	4.6 (0.0-19.44)	6.0 (0.0-19.44)	4.4 (0.0-14.6)
**BP180 NC15-Col11, median (range), index value**	5.7 (0.0-22.9)	6.1 (0.0-22.9)	3.2 (0.0-16.6)
**BP180 NC11-Col7, median (range), index value**	9.8 (0.0-70.6)	13.0 (2.2-70.6)	4.3 (0.0-28.0)
**BP180 NC7-Col4**			
**Median (range), index value**	19.1 (0.0-132.5)	28.5 (4.1-132.5)	5.4 (0.0-34.4)
**Positive cases, n (%)**	18 (69.2)	15 (83.3)	3 (37.5)
**BP180 NC4-NC1, median (range), index value**	8.3 (0.0-34.6)	9.3 (0.0-34.6)	5.68 (0.0-21.3)
**BP230**			
**Median (range), index value**	3.2 (1.9-115.8)	2.9 (1.9-115.8)	3.8 (1.2-93.9)
**Positive cases, n (%)**	6 (23.1)	3 (16.7)	3 (37.5)

Table S6. DPP4i-BP patient characteristics.

Characteristics	DPP4i-BP (N=60)
Age, median (range), years	78 (49-97)
Female sex, n (%)	20 (33.3)
DPP4i, n (%)	
Vildagliptin	15 (21.1)
Sitagliptin	11 (15.5)
Linagliptin	19 (26.8)
Teneligliptin	15 (21.1)
Alogliptin	9 (12.7)
Anagliptin	1 (1.4)
Trelagliptin	1 (1.1)
Full-length BP180	
Median (range), index value	68.9 (0.0-287.1)
Positive cases, n (%)	56 (93.3)
BP180 NC16A	
Median (range), index value	42.8 (0.9-198.1)
Positive cases, n (%)	40 (66.7)
BP180 Col15, median (range), index value	1.6 (0.0-34.4)
BP180 NC15-Col11, median (range), index value	2.2 (0.0-33.9)
BP180 NC11-Col7, median (range), index value	4.0 (0.0-70.6)
BP180 NC7-Col4	
Median (range), index value	20.9 (0.0-132.5)
Positive cases, n (%)	41 (68.3)
BP180 NC4-NC1, median (range), index value	3.4 (0.0-85.9)
BP230	
Median (range), index value	3.2 (0.0-115.8)
Positive cases, n (%)	14 (23.3)
BPDAI, median (range), score	
Erosions/Blisters	23 (1-80)
Urticaria/Erythema	3 (0-61)
Mucosa (Erosions/Blisters)	0 (0-20)
Total	29 (1-129)
Duration of DPP4i intake (N=35), median (range), years	21.0 (3.0-121.0)
Without oral PSL (N=56), positive cases (%)	30 (53.6)

Table S7. Comparison between DPP4i-BP NC7-Col4(+) NC16A(-) and NC16A(+).

	NC7-Col4(+) NC16A			
	**Negative (N=15)**	**Positive (N=26)**	**P-value**	**OR (95% CI)**
**Age, median (range), years**	79 (59-91)	79 (56-97)	0.8565	
**Female sex, n (%)**	3 (20.0)	11 (42.3)	0.1860	2.9 (0.6-11.3)
**Full-length BP180**				
**Median (range), index value**	93.9 (9.6-248.4)	78.0 (40.4-287.1)	0.5469	
**Positive cases (%)**	15 (100)	26 (100)	>0.9999	
**BP180 NC7-Col4**				
**Median (range), index value**	40.9 (10.3-88.9)	36.8 (10.6-132.5)	0.9893	
**Positive cases (%)**	15 (100)	26 (100)	>0.9999	
**BP230**				
**Median (range), index value**	2.9 (0.9-27.3)	3.4 (1.2-65.9)	0.5072	
**Positive cases (%)**	2 (13.3)	5 (19.2)	>0.9999	1.5 (0.3-8.6)
**DPP4i, n (%)**				
**Vildagliptin**	4 (23.5)	5 (17.2)	0.7068	0.7 (0.2-2.5)
**Sitagliptin**	1 (5.9)	3 (10.3)	>0.9999	1.8 (0.3-25)
**Linagliptin**	5 (29.4)	11 (37.9)	0.7500	1.5 (0.4-5.0)
**Teneligliptin**	5 (29.4)	5 (17.2)	0.4623	0.5 (0.1-2.1)
**Alogliptin**	2 (11.8)	4 (13.8)	>0.9999	1.2 (0.3-6.9)
**Anagliptin**	0 (0.0)	1 (3.4)	>0.9999	∞ (0.1-∞)
**Onset after DPP4i intake (N=11 vs. 10), median, months**	17.0 (3-59)	20.5 (10-64)	0.3578	
**BPDAI, median (range), score**				
**Erosions/Blisters**	9 (1-43)	27 (8-80)	**0.0017**	
**Urticaria/Erythema**	0 (0-6)	8 (0-61)	**0.0096**	
**Mucosa**	0 (0-5)	0 (0-20)	0.5368	
**Total**	13 (1-54)	37 (10-129)	**0.0001**	
**Without oral PSL (N=14 vs. 25), rates (%)**	71.4	36.0	**0.0484**	0.2 (0.1-0.9)

Table S8. Characteristics of DM+DPP4i patients.

	DM+DPP4i	
**Characteristics**	**Full-length BP180 (+) (N=24)**	**Full-length BP180 (-) (N=17)**
**Age, median (range), years**	74 (61-85)	66 (47-84)
**Female sex, n (%)**	11 (45.8)	7 (41.2)
**Full-length BP180 ELISA**		
**Median (range), index value**	9.2 (4.9-108.8)	0.0 (0.0-3.0)
**Positive rate, n (%)**	24 (100)	0 (0.0)

Data S1.

Spreadsheet showing BP180 HLA-class II peptide epitopes predicted by netMHCIIpan4.3 to bind with high affinity to *HLA-DQA1*05:05* and *HLA-DQB1*03:01*.
